# miR-125a-3p and miR-483-5p promote adipogenesis via suppressing the RhoA/ROCK1/ERK1/2 pathway in multiple symmetric lipomatosis

**DOI:** 10.1038/srep11909

**Published:** 2015-07-07

**Authors:** Ke Chen, Honghui He, Yanhong Xie, Liling Zhao, Shaoli Zhao, Xinxing Wan, Wenjun Yang, Zhaohui Mo

**Affiliations:** 1 Department of Endocrinology, Third Xiangya Hospital of Central South University, Changsha, Hunan, 410013, China

## Abstract

Multiple symmetric lipomatosis (MSL) is a rare disease characterized by symmetric and abnormal distribution of subcutaneous adipose tissue (SAT); however, the etiology is largely unknown. We report here that miR-125a-3p and miR-483-5p are upregulated in the SAT of MSL patients, promoting adipogenesis through suppressing the RhoA/ROCK1/ERK1/2 pathway. TaqMan microRNA (miR) array analysis revealed that 18 miRs were upregulated in the SAT of MSL patients. Transfection of human adipose-derived mesenchymal stem cells (hADSCs) with the individual agomirs of these 18 miRs showed that miR-125a-3p and miR-483-5p significantly promoted adipogenesis. A dual-luciferase assay showed that RhoA and ERK1 were the targets of miR-125a-3p and miR-483-5p, respectively. Moreover, transfection of hADSCs with mimics of miR-125a-3p and miR-483-5p resulted in a pronounced decrease of ERK1/2 phosphorylation in the nucleus; conversely, transfection of hADSCs with inhibitors of miR-125a-3p and miR-483-5p led to a significant increase of ERK1/2 phosphorylation in the nucleus. Most importantly, we found that miR-125a-3p and miR-483-5p promoted *de novo* adipose tissue formation in nude mice. These results demonstrated that miR-125a-3p and miR-483-5p coordinately promoted adipogenesis through suppressing the RhoA/ROCK1/ERK1/2 pathway. Our findings may provide novel strategies for the management and treatment of MSL or obesity.

Multiple symmetric lipomatosis (MSL) is a rare disease characterized by a rapid progression of multiple, symmetrical, and nonencapsulated adipose tissue in the face, neck, shoulder, back, and abdomen^1^. The incidence rate is approximately 1 in 25,000, and around 400 cases have been reported since the initial description by Brode in 1846. Up to 90% of MSL patients also suffer from chronic alcoholism^2^, whereas the underlying molecular mechanism is still enigmatic and there is no effective treatment for MSL.

The distinctive pathological characteristic of MSL is the substantial adipocyte accumulation in the subcutaneous adipose tissue (SAT), but most patients do not demonstrate dysfunction of glucose or lipid metabolism, as reported previously in one case by us^3^,^4^. Chronic alcohol ingestion or A8344G mutation of mitochondrial DNA has been considered as an important risk factor for MSL^5^,^6^,^7^. However, the molecular mechanism of the enhanced differentiation of adipocytes in MSL remains to be determined.

MicroRNAs (miRs) are small non-coding RNAs of 19 to 25 nucleotides that regulate target gene expression and participate in adipocyte differentiation^8^,^9^,^10^ For instance, miR-30 inhibits the differentiation of mesenchymal stem cells (MSCs) to preadipocytes^11^; while, the miR-17-92 cluster accelerates preadipocyte clonal expansion through Rb2/p130^12^, miR-143 and miR-375, respectively, enhance adipogenesis through the ERK5 and ERK1/2 pathways, two important regulators of the mitogen-activated protein kinase (MAPK) signaling pathway that suppresses adipogenesis^13^,^14^.

Accumulating evidence suggests that miR-125a plays an important role during adipogenesis^15^,^16^. Importantly, Ras homolog family member A (RhoA), a small GTPase that plays key roles in adipogenesis, has been reported as a target gene of miR-125a-3p^17^. In human mesenchymal stem cells (hMSCs) as well as mouse adipose-derived stromal cells (mASCs), overexpression of dominant-negative RhoA induced hMSCs or mMSCs to adipocytes; whereas constitutively active RhoA or Rho-associated kinase (ROCK), an effector of RhoA, led to osteogenesis^18^,^19^. Similarly, knockdown of RhoA with RNAi or pharmacological inhibition of RhoA or ROCK in preadipocytes promoted adipogenesis in mouse 3T3-L1 cells; in contrast, ectopic overexpression of RhoA or treatment with the RhoA agonist lysophosphatidic acid inhibited adipogenesis in mouse 3T3-L1 cells^20^,^21^. Thus, the RhoA/ROCK pathway is a “switch” not only in terms of the stage of stem cells to preadipocytes but also during the process of preadipocytes to mature adipocytes. Targeting RhoA indicates a key role of miR-125a-3p during adipogenesis.

It is widely accepted that ERK1 inhibits adipogenesis via suppressing its downstream target gene peroxisome proliferator-activated receptor gamma (PPARγ), which is a critical nuclear transcription factor of adipogenesis^22^,^23^,^24^. It has been reported that miR-483-3p regulates adipogenesis^25^ and miR-483-5p targets ERK1^26^. Since RhoA promotes the activation of ERK signaling via ROCK^27^, we hypothesized that miR-125a-3p and miR-483-5p may jointly promote adipogenesis in MSL via the RhoA/ROCK/ERK1/2 pathway.

In this study, we first systematically investigated the expression profile of miRs in SAT between MSL patients and control subjects. Next, we verified the regulation of adipogenesis by miR-125a-3p and miR-483-5p in hADSCs by overexpression or downregulation of miR-125a-3p and miR-483-5p, and analyzed RhoA and ERK1 by luciferase reporter assays. Then, we explored the interactions of miR-125a-3p and miR-483-5p on the RhoA/ROCK/ERK1/2 pathway. Finally, we observed adipogenesis of nude-mouse subcutaneous hADSCs following transfection of miR-125a-3p and miR-483-5p.

## Methods

### Sample selection and preparation

Three male MSL and three male control subjects were recruited in this study. Three control subjects had no diabetes, malignant tumors, acute infectious disease, and smoking history. Anthropometric and metabolic characteristics were evaluated according to our previously study^4^. The SAT was obtained from the right upper quadrant of the abdomen for all subjects. The study protocol was approved by the Human Ethical Review Committee of the Third Xiangya Hospital of Central South University, Changsha, China; and all subjects signed the informed written consent. All methods used in this study were carried out in accordance with the approved guidelines.

### RNA extraction and TaqMan MicroRNA array analysis

Total RNA was isolated with a TRIzol RNA extraction kit (Life Technologies, Carlsbad, CA, USA). The miR enrichment was performed with an mirVana miRNA Isolation Kit and converted to cDNA by a TaqMan MicroRNA Reverse Transcription Kit (Applied Biosystems, Life Technologies, USA), according to the manufacturer’s instructions. The reverse transcription products were used with the TaqMan Human MicroRNA Array A+B cards set v3.0 (Applied Biosystems, Life Technologies, USA) to detect 754 human miRs. miRs expression fold changes were calculated by the 2^−ΔΔCT^ method. Kyoto Encyclopedia of Genes and Genomes (KEGG) pathway analysis was performed according to the method described by Wen *et al.*^28^.

### hADSC isolation and differentiation

hADSCs were isolated from the abdominal SAT of two control patient. Ten gram tissue was washed three times with D-Hank’s solution (Gibco, Life Technology, China), cut into 1-mm × 1-mm pieces, and digested with collagenase I (Gibco, Life Technology, China) for 1 h at 37 °C. The cells were collected by centrifugation at 150 × *g* for 10 min, then lysed in erythrocyte lysis buffer (0.154 M NH_4_Cl, 10 mM KHCO_3_, and 0.1 mM EDTA) for 10 min, and filtered by a nylon mesh. The supernatant was collected and centrifuged again. The pelleted cells were cultured in DMEM/F12 medium (Gibco, Life Technology, China) supplemented with 10% fetal bovine serum (Gibco, Life Technology, Australia). After 24 h, the medium was replaced with fresh medium. The cells were cultured for 4–7 passages for experiments. To induce hADSC differentiation, over-confluent hADSCs were cultured in inducing medium (DMEM/F12 supplemented with 1 μM dexamethasone, 10 μM insulin, 0.5 mM isobutylmethylxanthine (IBMX), and 200 μM indomethacin (all provided by Sigma, St. Louis, MO, USA). The medium was replaced every 2 days. For hADSC self-differentiation, cells were maintained in DMEM/F12 medium without the differentiation-inducing reagents. We repeated all experiments by two kinds of hADSCs that were isolated from two control patients.

### Quantitative real-time polymerase chain reaction (PCR)

The miR reverse transcription was conducted using a Reverse Transcription Kit (Life Technologies, Carlsbad, CA, USA). 0.5 μM miRNA PCR primers (RiboBio, Guangzhou, China) were used for PCR amplification. Real-time PCR was performed with TaqMan Universal PCR Master Mix with No AmpErase UNG (Applied Biosystems, Life Technologies, USA) on an Applied Biosystems 7900HT System (Applied Biosystems, Life Technologies, USA). Raw Ct values were calculated using the RQ Manager software v1.2 (Applied Biosystems, Life Technologies, USA) with automatic baseline and threshold settings. The miRs were normalized to the level of U6 snRNA.

### Cell transfection

The miR mimics, mimic-NC, inhibitor, inhibitor-NC, cholesterol-conjugated 2′-*O*-methyl-modified mimics (agomir), agomir-NC, antagomir, and antagomir-NC were synthesized by RiboBio (RiboBio, Guangzhou, China), pCEP4L-ERK1 and pCEP4L-NC plasmids were purchased from Addgene (Cambridge, MA, USA). When the hADSCs were approximately 30–40% confluent, the mimic or agomir (100 nM), inhibitor or antagomir (200 nM) miRs were transfected with Lipofectamine 2000 (Invitrogen, Carlsbad, CA, USA). After 72 h, the cells were subjected to the process of induction of differentiation.

### Oil red O staining

The cells were washed two times in D-Hank’s solution, fixed in 4% formaldehyde for 30 min, and washed three times with water. Then, the cells were stained with Oil red O (Sigma, St. Louis, MO, USA) for 15 min. Following three washes in water, the lipid droplets were observed and photographed under a microscope (TE2000-E; Nikon, Japan).

### Vector construction

The human RhoA and ERK1 3′-UTR containing the predicted binding site sequences were amplified by RT-PCR with the following primers: RhoA forward primer: 5′-CTTGACTCGAGAACCTTGCTGCAAGCACAG-3′, reverse primer: ATTGCGGCCGCTGCCTTTATTCTATTAGTAG-3′; ERK1 forward primer: 5′-CTTGACTCGAGCTCTGGCAGTTCTGGAATGG-3′, reverse primer: 5′- ATTGCGGCCGCATATTAGACGGGTCAGG-3′. The XhoI and NotI restriction sites are underlined. The amplified fragments were inserted into the dual luciferase plasmid pmiR-RB-REPORT™ (RiboBio, Guangzhou, China). Mutation of the seed target sequence of the human RhoA 3′-UTR from CUCACCUG to GACACCUG, and ERK1 3′-UTR from CCCGUCU to GGGGUCU was performed using a Quickchange site-directed mutagenesis kit (Agilent Technologies, Edinburgh, UK).

### Dual-luciferase reporter assay

The 293T cells were cultured in 96-well plates in DMEM supplemented with 10% fetal bovine serum at 37 °C with 5% CO_2_. When the cells reached 70–80% confluence, the wild-type (WT) or mutation-type (MT) RhoA and ERK1 3′-UTR plasmids were cotransfected with miR-125a-3p or miR-483-5p mimics (100 nM) or negative control (NC) mimics with Lipofectamine 2000. After 48 h, the cells were washed twice with PBS and lysed with passive lysis buffer (PLB) before dual-luciferase reporter assay agents were added (Beyotime, Haimen, China). Firefly and renilla luciferase activities were measured with the Dual-Glo luciferase assay system (Promega, Madison, WI, USA). All experiments were performed three times.

### Western blot

Cells were lysed on ice in radioimmunoprecipitation assay (RIPA) buffer with protease inhibitors (Beyotime, Haimen, China). Total and nuclear proteins were extracted according to the manufacturer’s instructions (Beyotime, Haimen, China). Protein samples (50 μg) were separated on a 10% SDS-PAGE gel and transferred onto a polyvinylidene difluoride membrane (Millipore, Bedford, MA, USA). The membranes were blocked in TBST containing 5% fat-free milk at room temperature for 1 h. After washing with TBST, the membranes were probed with primary antibodies against C/EBPα, FABP4, PPARγ, RhoA, ROCK1, ROCK2, ERK1/2, β-actin, PCNA (all Proteintech, Wuhan, China), and p-ERK1/2 (Cell Signaling Technology, Danvers, MA, USA). The membranes were washed in TBST and incubated with secondary antibody (Proteintech, Wuhan, China). The signals were developed by ECL reagents (Beyotime, Haimen, China).

### Lentiviral construction and transfection

To generate hsa-miR-125a and hsa-miR-483 lentiviral expression plasmids, 268-bp and 273-bp sequences containing pre-hsa-miR-125a or pre-hsa-miR-483 were synthesized and cloned into lentiviral expression vector pGC-FU (GeneChem, Shanghai, China), respectively. After sequence confirmation by DNA sequencing, the viruses were packaged in 293T cells after cotransfection with pHelper 1.0 and pHelper 2.0 Packing Plasmid (GeneChem, Shanghai, China) using Lipofectamine 2000. To obtain stable lentivirus-infected cell lines, the cells were plated at 30–40% confluence and lentiviral vector with 2 mg/ml polybrene (GeneChem, Shanghai, China) resolved in serum-free medium was transfected. After 16 h, the medium was replaced with fresh complete medium. 72 h later, the transfection efficiency was verified by fluorescence microscopy.

### De novo adipogenesis *in vivo*

To induce *de novo* adipose tissue, hADSCs were transfected with one of the following lentiviruses: negative control miR (LV-NC), hsa-miR-125a, hsa-miR-483, or hsa-miR-125a +483. A total of 1.5 × 10^7^ transfected hADSCs were harvested and resuspended in 200 μl of PBS. The cells or PBS were injected subcutaneously in the backs of 6-week-old male athymic Balb/c nude mice (Chinese Academy of Sciences, Shanghai, China). The mice were fed with a normal diet on a 12-h day/night cycle for 5 weeks. Five mice from each group were sacrificed. The *de novo* adipose tissues were removed and weighed. Some samples were stained with hematoxylin and eosin. Adipoctye areas were measured and analysed by ImageJ 1.48μ digital imaging processing software. The other samples were used for real-time PCR or western blot analyses.

All animal experiments were approved by the Human Ethical Review Committee of the Third Xiangya Hospital of Central South University, Changsha, China, and performed in accordance with the NIH Guide for the Care and Use of Laboratory Animals (1996).

### Statistical analyses

All the results are presented as means ± standard deviation. Two groups were compared by the unpaired Student’s t-test, and multiple groups were analyzed by one-way analysis of variance. Statistical significance was defined as a *P* value <  0.05.

## Results

### Some miRs are upregulated in MSL patients

**Figure 1:**
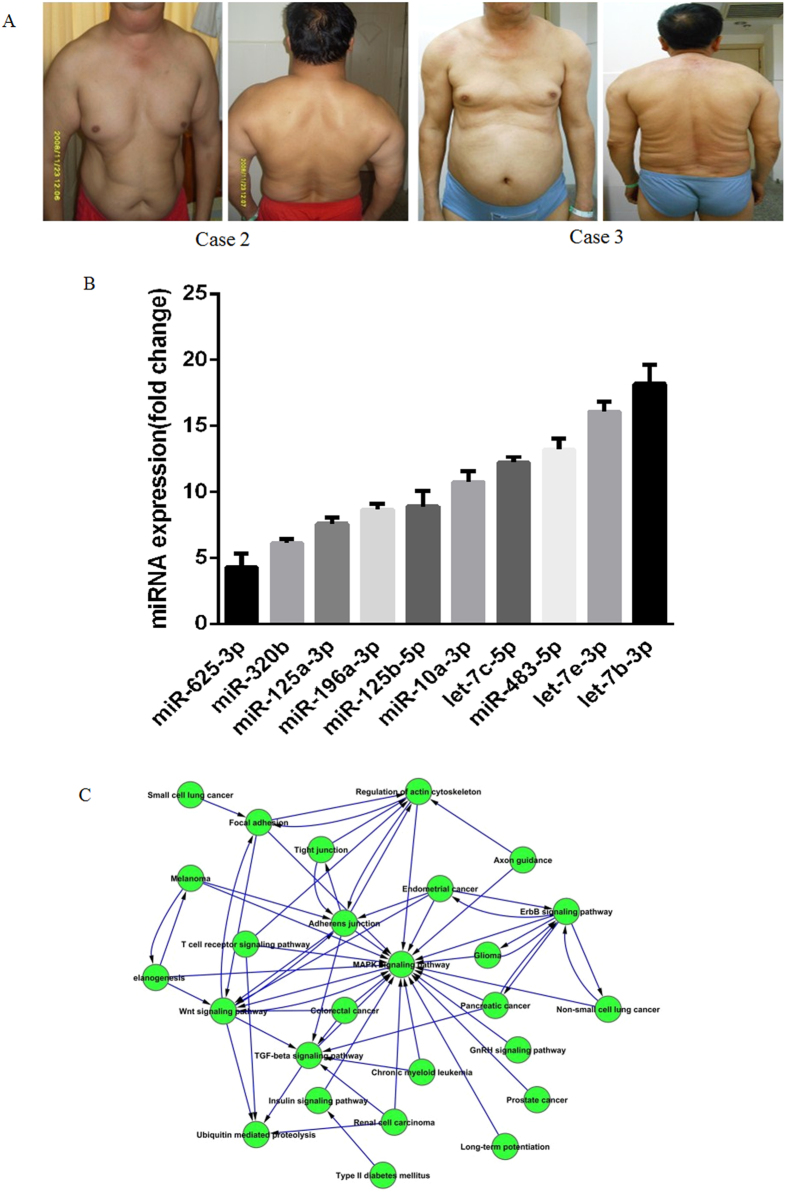
Photographs of two MSL patients and TaqMan miR array analysis of SAT. (**A**) There was symmetrical SAT accumulation in the neck, upper arms, bilateral shoulders, upper thorax, back, and abdomen. (**B**) Relative expression of ten miRs were verified by quantitative real-time PCR (n = 3). (**C**) KEGG pathway analysis of the upregulated miRs in the SAT of MSL patients.

All three MSL patients [including one case we have reported previously^4^] were addicted to alcohol for 15, 10, and 8 years and had gradually increased SAT accumulation for 7, 5, and 3 years, respectively. They all showed symmetrical and substantial SAT accumulation in the neck, upper arms, bilateral shoulders, upper thorax, back, and abdomen (Fig. 1A). In order to identify the differential expression of miRs in SAT between MSL patients and controls, the miR expression profiles were detected by TaqMan miR arrays. Some anthropometric and metabolic characteristics were described in Table 1. Among the 754 human miRs, 18 miRs were upregulated in the SAT of MSL patients compared to that of the control group; and no miR was significantly downregulated in the SAT of MSL patients (Table 2). Ten of the 18 miRs were further verified by miR real-time PCR (Fig. 1B). KEGG pathway analysis revealed that some adipogenesis-related pathways including Wnt, TGF-β, actin cytoskeleton, and especially MAPK were significantly enriched (Fig. 1C). These results imply that miRs may play important roles during adipogenesis in MSL patients.

### miR-125a-3p and miR-483-5p significantly promote adipogenesis in hADSCs

**Figure 2:**
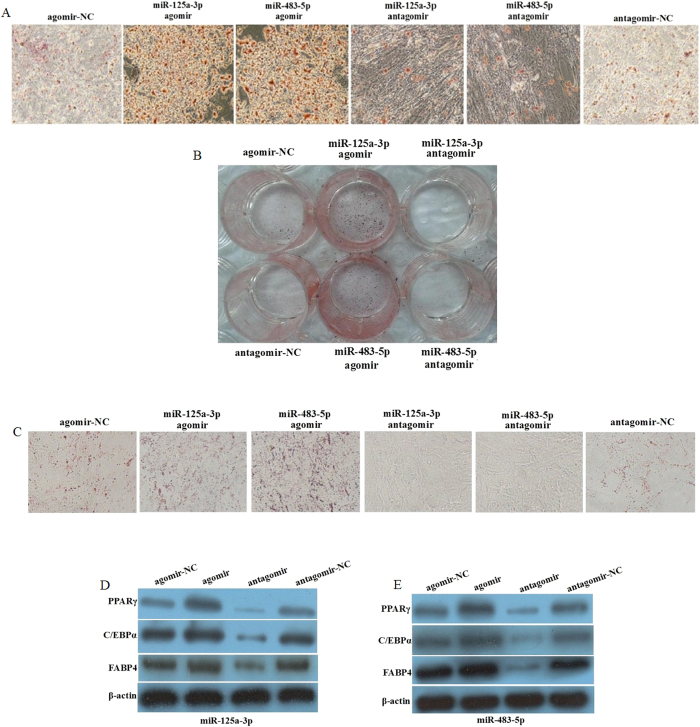
miR-125a-3p and miR-483-5p regulate adipogenesis in hADSCs. hADSCs were isolated from the abdominal SAT of two control patient respectively, and we repeated all experiments by two kinds of hADSCs. The hADSCs were transfected with the miR-125a-3p or miR-483-5p agomir, agomir-NC (100 nM), antagomir-NC, or antagomir (200 nM), respectively. After 72 h, (**A**) The hADSCs were induced differentiation for 12 days, lipid droplet accumulation stained by Oil red O was observed under a microscope. (**B**) The hADSCs were induced differentiation for 12 days, lipid droplet accumulation stained by Oil red O was observed by a camera (no magnification). (**C**) The hADSCs underwent self-differentiation for 12 days, lipid droplet accumulation stained by Oil red O was observed under a microscope. (**D**,**E**) The hADSCs were induced differentiation for 12 days, the protein levels of PPARγ, C/EBPα, and FABP4 were detected by Western blot. β-Actin served as a loading control. All measurements were preformed by three independent experiments.

To investigate which miRs may promote adipogenesis, all 18 miRs with agomir were respectively transfected into hADSCs, which were further induced to mature adipocytes or underwent self-differentiation for 12 days. We found that transfection with miR-125a-3p or miR-483-5p significantly promoted lipid droplet accumulation in hADSCs that were induced to mature adipocytes. Conversely, inhibition of miR-125a-3p or miR-483-5p with the corresponding antagomir markedly decreased lipid droplet accumulation (Fig. 2A,B). The same results were observed in the hADSCs undergoing self-differentiation for 12 days (Fig. 2C). Next, after each group was induced to mature adipocytes for 12 days, C/EBPα, PPARγ, and FABP4 were upregulated by the agomir of miR-125a-3p or miR-483-5p; while they were downregulated by the antagomir of miR-125a-3p or miR-483-5p (Fig. 2D,E). These results demonstrated that miR-125a-3p and miR-483-5p promote adipogenesis in hADSCs.

### RhoA and ERK1 are the target genes of miR-125a-3p and miR-483-5p, respectively

**Figure 3:**
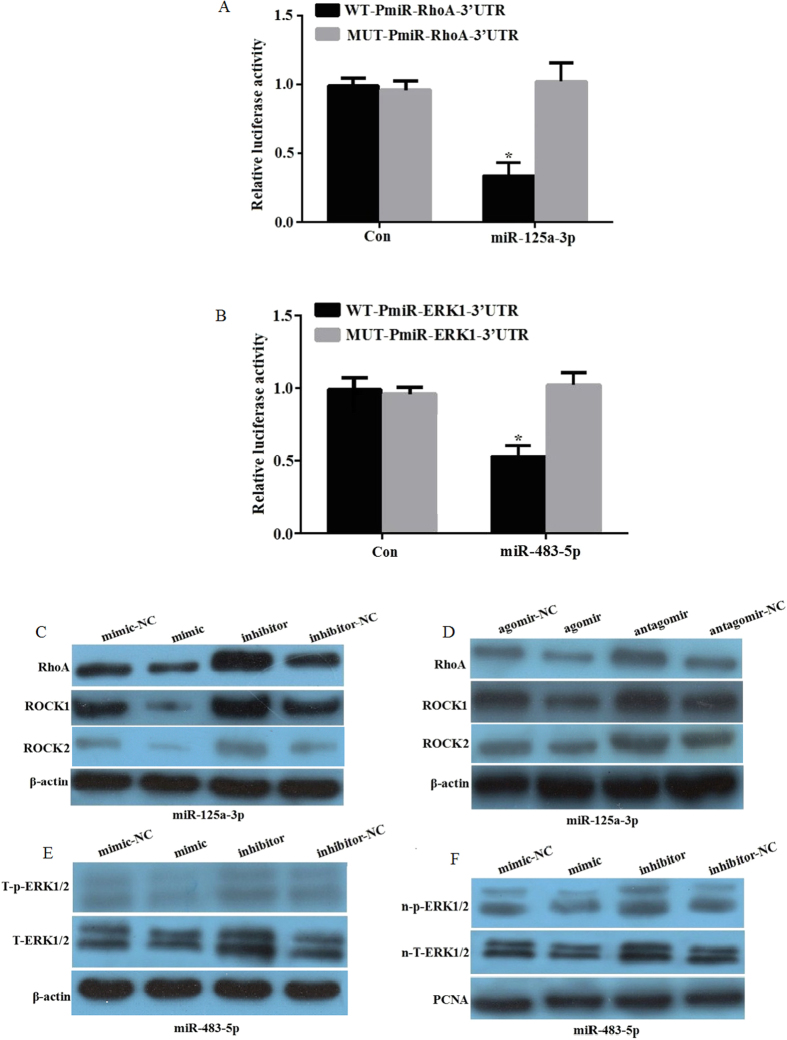
RhoA and ERK1 are the respective target genes of miR-125a-3p and miR-483-5p. (**A**) Dual-luciferase reporter plasmid containing human wild-type (WT-Pmir-RhoA-3′-UTR) or mutant RhoA-3′-UTR (MUT-Pmir-RhoA-3′-UTR) was cotransfected with the miR-125a-3p mimic (100 nM) or nontarget control (NC), respectively. Firefly and renilla luciferase activity was determined and normalized to firefly luciferase activity. (**B**) Dual-luciferase reporter plasmid containing human wild-type (WT-Pmir-ERK1-3′-UTR) or mutant RhoA-3′-UTR (MUT-Pmir-ERK1-3′-UTR) was cotransfected with the miR-483-5p mimic (100 nM) or nontarget control (NC), respectively. Firefly and renilla luciferase activity was determined and normalized to firefly luciferase activity. (**C**) hADSCs were transfected with the miR-125a-3p mimic, inhibitor, and controls for 72 h. Total protein was extracted for immunoblotting of RhoA and ROCK1. (**D**) hADSCs were transfected with the miR-125a-3p agomir, antagomir, and controls for 72 h, and induced to mature adipocytes for 12 days. Total protein was extracted for immunoblotting of RhoA and ROCK1. (**E**) hADSCs were transfected with the miR-483-5p mimic, inhibitor, and controls for 72 h. Whole cell protein was extracted for immunoblotting to detect total ERK1/2 (T-ERK1/2) and total phosphorylated ERK1/2 (T-p-ERK1/2). (**F**) hADSCs were transfected with the miR-483-5p mimic, inhibitor, and controls for 72 h. Whole nuclear protein was extracted to detect nuclear total ERK1/2 (n-T-ERK1/2) and nuclear phosphorylated ERK1/2 (n-p-ERK1/2).*p < 0.05, compared to 293T cells transfected with miR control mimics. All measurements were preformed by three independent experiments.

To further support previous observations^18^,^27^, we designed different mutant site sequences in the 3′-UTR of RhoA and ERK1 and verified the targeting of RhoA and ERK1 by miR-125a-3p and miR-483-5p, respectively. We found that the miR-125a-3p mimic significantly downregulated the luciferase activity of WT-PmiR-RhoA-3′-UTR compared to the control mimic. In sharp contrast, the miR-125a-3p mimic had no effect on the luciferase activity of MUT-PmiR-RhoA-3′-UTR (Fig. 3A). Similar results were observed in miR-483-5p and ERK1 (Fig. 3B). Next, we transfected hADSCs with the miR-125a-3p or miR-483-5p mimic or inhibitor for 72 h and determined the protein levels of RhoA or ERK1/2 by immunoblotting. The results showed that the miR-125a-3p mimic significantly downregulated RhoA and its downstream factor ROCK1 and ROCK2; conversely, the miR-125a-3p inhibitor significantly upregulated RhoA, ROCK1 and ROCK2 (Fig. 3C). Similar results were observed in mature adipocytes after hADSCs were transfected with the miR-125a-3p agomir or antagomir for 72 h and induced for 12 days (Fig. 3D). Total ERK1/2 (T-ERK1/2) and total phosphorylated ERK1/2 (T-p-ERK1/2) from whole cell protein lysate were downregulated by the miR-483-5p mimic, while they were upregulated by the miR-483-5p inhibitor in hADSCs following transfection for 72 h (Fig. 3E). Similar results were found for nuclear total ERK1/2 (n-T-ERK1/2) and nuclear phosphorylated ERK1/2 (n-p-ERK1/2) in whole nuclear protein lysate (Fig. 3F). We did not investigate the protein expression T-p-ERK1/2, n-T-ERK1/2 and n-p-ERK1/2 due to their very low expression in mature adipoctye. These results clearly demonstrated that RhoA and ERK1 are the direct target genes of miR-125a-3p and miR-483-5p, respectively.

### miR-125a-3p and miR-483-5p are upregulated during the differentiation of hADSCs, and target proteins are downregulated in MSL

**Figure 4:**
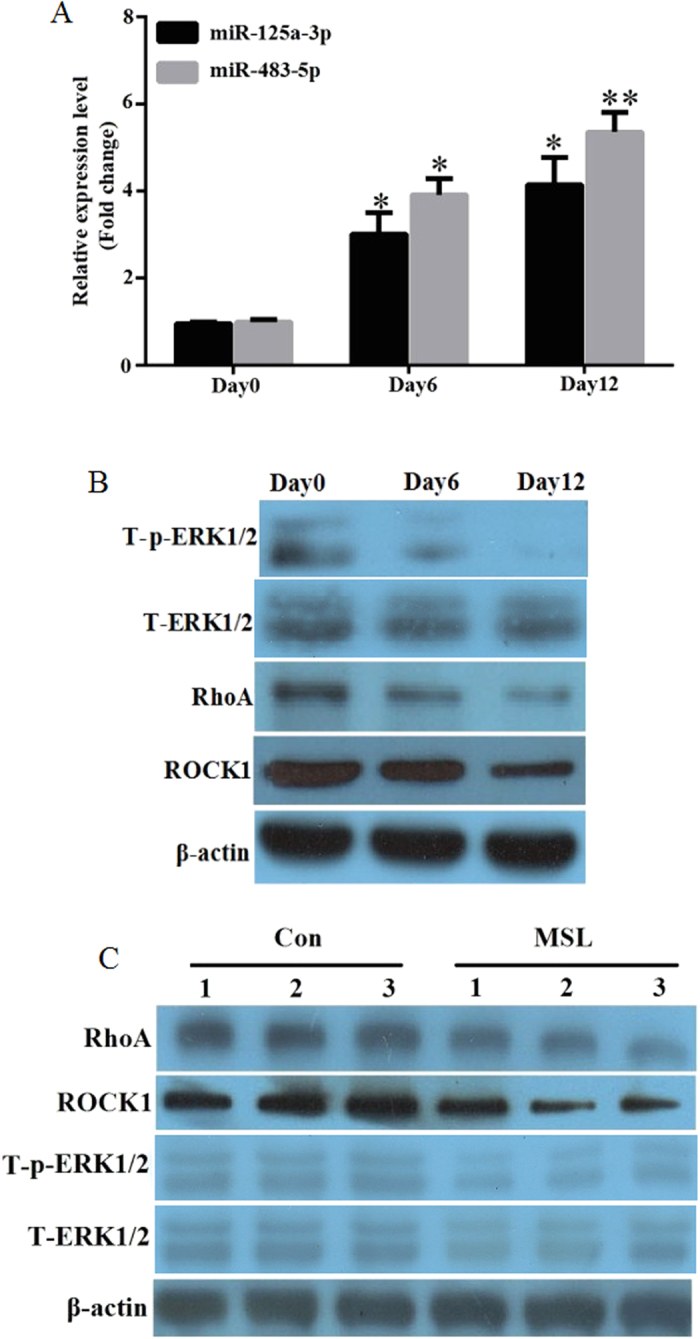
The expression of miR-125a-3p and miR-483-5p are increased with the induction of hADSCs. (**A**) The hADSCs were induced to mature adipocytes for 12 days. The miR-125a-3p and miR-483-5p expression levels were detected by quantitative real-time PCR at days 0, 6, and 12. (**B**) The hADSCs were induced to mature adipocytes for 12 days. Total protein was extracted for immunoblotting of RhoA, ROCK1, total ERK1/2 (T-EKR1/2), and phosphorylated ERK1/2 (T-p-EKR1/2) by western blot. (**C**) The expression of RhoA, ROCK1, T-EKR1/2, and T-p-EKR1/2 in the adipose tissues of MSL patients (n = 3) and controls (n = 3) were determined by western blot. *p < 0.05, **p < 0.01 in comparison to relative expression levels at day 0.

To investigate the expression tendency of miR-125a-3p and miR-483-5p during hADSC differentiation, we used real-time PCR to detect the miR-125a-3p and miR-483-5p expression at days 0, 6, and 12 during the induction to mature adipocytes. Relative to day 0, approximately 3-4- and 4-6-fold increases of miR-125a-3p and miR-483-5p expression were observed at day 6 and day 12 (Fig. 4A), respectively. Similarly, RhoA, ROCK1, and T-p-ERK1/2 were gradually downregulated with the induction time of hADSCs, however, T-ERK1/2 had no change (Fig. 4B). We further assessed the target gene expression in the SAT of the MSL patients and found that RhoA, ROCK1, T-ERK1/2, and T-p-ERK1/2 were all downregulated in the MSL patients compared to that of the controls (Fig. 4C). These results imply that miR-125a-3p and miR-483-5p may play an important role in MSL adipogenesis.

### miR-125a-3p and miR-483-5p jointly regulate the RhoA/ROCK1/ERK1/2 pathway

**Figure 5:**
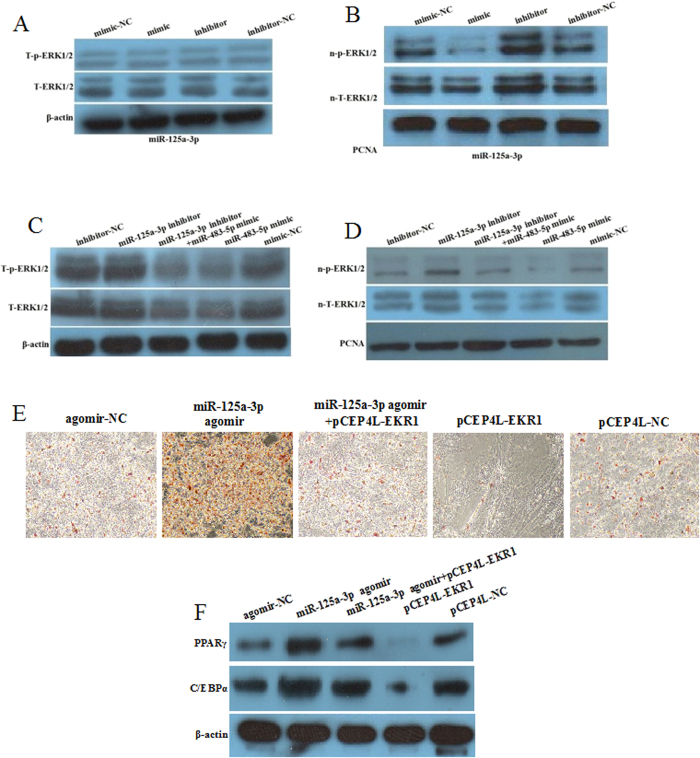
miR-125a-3p and miR-483**-**5p synergistically regulate the RhoA/ROCK1/ERK1/2 pathway. (**A**) hADSCs were transfected with the miR-125a-3p mimic, inhibitor, and controls for 72 h. Total protein was extracted for immunoblotting of total ERK1/2 (T-EKR1/2) and phosphorylated ERK1/2 (T-p-EKR1/2). (**B**) hADSCs were transfected with the miR-125a-3p mimic, inhibitor, and controls for 72 h. Nuclear protein was extracted for immunoblotting of n-T-ERK1/2 and n-p-ERK1/2. (**C**) hADSCs were transfected with the miR-125a-3p inhibitor, miR-483-5p mimic, miR-125a-3p inhibitor plus miR-483-5p mimic, or controls for 72 h. Total protein was extracted for immunoblotting of T-EKR1/2 and T-p-EKR1/2. (**D**) hADSCs were transfected with the miR-125a-3p inhibitor, miR-483-5p mimic, miR-125a-3p inhibitor plus miR-483-5p mimic, or controls for 72 h. Nuclear protein was extracted for immunoblotting of n-T-ERK1/2 and n-p-ERK1/2. β-actin and PCNA served as loading controls. (**E**) hADSCs were transfected with miR-125a-3p agomir, pCEP4L-ERK1, miR-125a-3p agomir plus pCEP4L-ERK1, agomir-NC or pCEP4L-NC and induced for differentiation for 12days. Lipid droplet accumulation stained by Oil red O was observed under a microscope. (**F**) The hADSCs were induced differentiation for 12 days, the protein levels of PPARγ and C/EBPα were detected by western blot. β-Actin served as a loading control.

To determine whether miR-125a-3p and miR-483-5p jointly regulate the activity of the RhoA/ROCK1/ERK1/2 pathway, we first detected the expression of T-ERK1/2, T-p-ERK1/2, n-T-ERK1/2, and n-p-ERK1/2 following transfection of hADSCs with the mimic or inhibitor of miR-125a-3p. Transfection with either the mimic or inhibitor of miR-125a-3p did not alter the T-ERK1/2 or T-p-ERK1/2 expression levels (Fig. 5A). However, the miR-125a-3p mimic apparently decreased the levels of n-T-ERK1/2 and n-p-ERK1/2; while the miR-125a-3p inhibitor increased the levels of n-T-ERK1/2 and n-p-ERK1/2 (Fig. 5B). Next, we cotransfected the miR-125a-3p inhibitor and miR-483-5p mimic to test ERK1/2 expression in hADSCs. In comparison to the control and miR-125a-3p inhibitor, cotransfection with the miR-125a-3p inhibitor and miR-483-5p mimic resulted in decreased expression of T-ERK1/2 and T-p-ERK1/2 to the levels of the miR-483-5p mimic group (Fig. 5C). Moreover, n-T-ERK1/2 and n-p-ERK1/2 were downregulated in the cotransfected group compared to that of the miR-125a-3p inhibitor group while upregulated compared to that of the miR-483-5p mimic group (Fig. 5D). Next, we transfected hADSCs with miR-125a-3p agomir or pCEP4L-ERK1, or cotransfected with the miR-125a-3p agomir and pCEP4L-ERK1 and induced differentiation for 12 days. miR-125a-3p agomir significantly promoted adipogenesis, and pCEP4L-ERK1 almost completely inhibited adipogenesis; however, cotransfection group partially restored adipogenesis (Fig. 5E). Similar protein expression of PPARγ and C/EBPα were observed in mature adipogenesis and cotransfection group increased PPARγ and C/EBPα compared to pCEP4L-ERK1 (Fig. 5F). These results suggested that miR-125a-3p and miR-483-5p might jointly regulate the activity of the RhoA/ROCK1/ERK1/2 signaling pathway.

### miR-125a-3p and miR-483-5p promote *de novo* adipose tissue formation in nude mice

**Figure 6:**
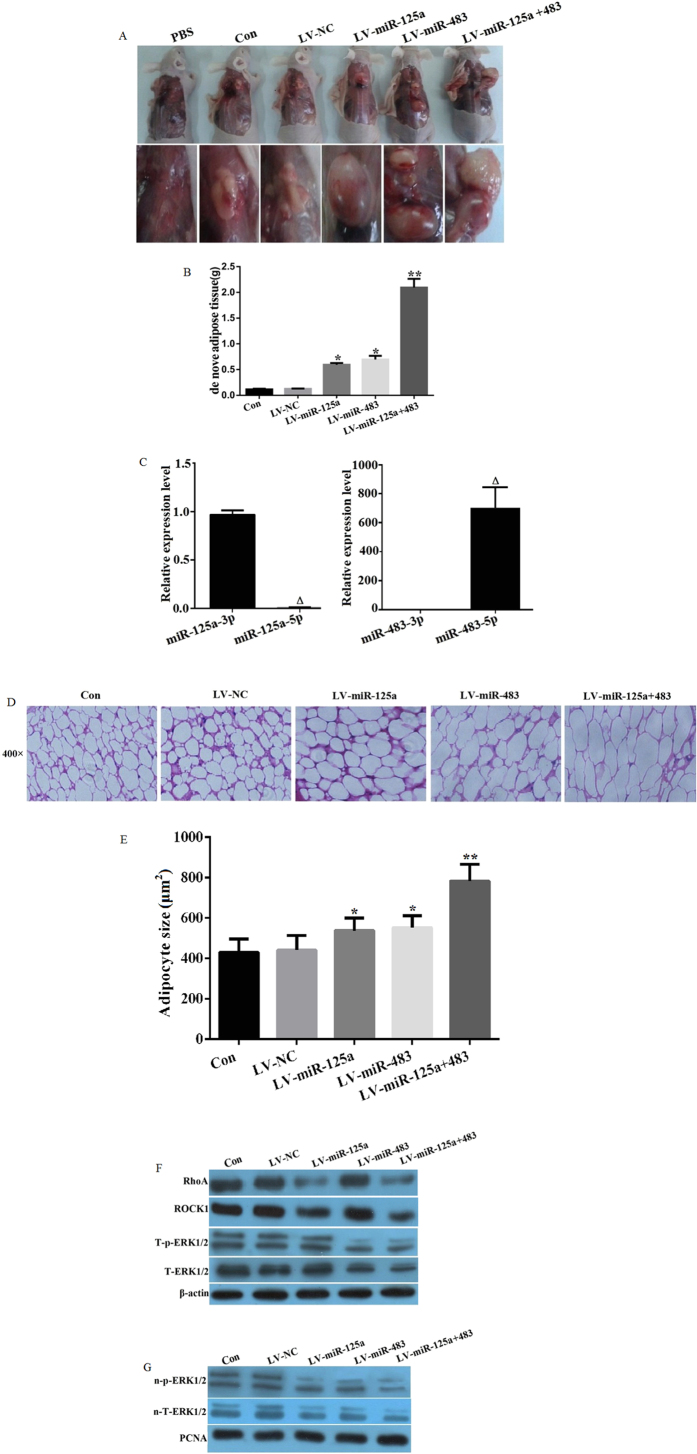
miR-125a-3p and miR-483-5p regulate RhoA/ROCK1/ERK1/2 signaling and promote mouse *de novo* adipogenesis. hADSCs were transfected with a lentiviral vector containing pre-miRs of miR-125a (LV-miR-125a), miR-483 (LV-miR-483), or negative control miR (LV-NC) and transplanted to the back subcutaneous tissues of nude mice with the transfected hADSCs or non-tranfected hADSC as control group (Con) or injection of PBS. After 5 weeks of self-differentiation, (**A**) *De novo* adipose tissue formation was observed (n = 5); (**B**) The weights of the *de novo* adipose tissues were measured (n = 5); (**C**) The expression levels of miR-125a-3p/5p and miR-483-3p/5p in *de novo* adipose tissue were detected by real-time PCR (n = 5); (**D**) The *de novo* adipose tissue was stained to observe the histology (n = 5); (**E**) Adipoctye size were analyzed by ImageJ software (**F**) The protein expression levels of RhoA, ROCK1, total ERK1/2 (T-EKR1/2), and phosphorylated ERK1/2 (T-p-EKR1/2) were analyzed by western blot; and (**G**) Total nuclear ERK1/2 (n-T-ERK1/2) and p-ERK1/2 (n-p-ERK1/2) were analyzed by western blot. All measurements were preformed by three independent experiments. *p < 0.05, **p < 0.01 compared to control or LV-NC group. Δp < 0.01 compared to miR-125a-3p or miR-483-3p group.

To investigate the roles of miR-125a-3p and miR-483-5p in regulating adipogenesis *in vivo*, we transfected hADSCs with lentivirus containing pre-miRs of miR-125a (LV-miR-125a), miR-483 (LV-miR-483), LV-miR-125a + 483 or negative control miR (LV-NC). Almost 100% transfection efficiency was verified by fluorescence microscopy (data not shown). Each group of cells were transplanted the back subcutaneous tissues of nude mice with the transfected hADSCs or non-tranfected hADSC as control group (Con) or injection of PBS. After 5 weeks of self-differentiation, we observed the *de novo* adipose tissue formation. No *de novo* adipose tissue formation was observed in the PBS group. Interestingly, capsular *de novo* adipose tissues were found in the LV-miR-125a, LV-miR-483, and cotransfection group; however, no capsular formation was seen in the Con or LV-NC groups. The *de novo* adipose tissue formation in LV-miR-125a, LV-miR-483 and cotransfection group were much larger than that of the Con or LV-NC groups, especially in cotransfection group (Fig. 6A,B). In the *de novo* adipose tissues of LV-miR-125a or LV-miR-483 group, we found that miR-125a-3p and miR-483-5p expression was significantly higher than miR-125a-5p and miR-483-3p expression (Fig. 6C). In addition, adipose tissue staining showed cell size of miR-125a or miR-483 was bigger than Con or LV-NC group, but smaller than cotransfection group (Fig. 6D,E). Lastly, we assessed the activity of the RhoA/ROCK1/ERK1/2 pathway. In the LV-miR-125a group, there was no apparent alteration of the T-ERK1/2 and T-p-ERK1/2 levels, but the RhoA and ROCK1 levels were decreased. In contrast, T-ERK1/2 and T-p-ERK1/2 were both decreased in the LV-miR-483 and cotransfection group compared to that of the control, LV-NC, or LV-miR-125a groups, while LV-miR-483 did not affect RhoA and ROCK1 levels (Fig. 6F). n-T-ERK1/2 and n-p-ERK1/2 were downregulated in the LV-miR-125a and LV-miR-483 groups, and even greater downregulation was observed in the cotransfection group compared to the LV-miR-125a and LV-miR-483 groups (Fig. 6G). Our results suggest that miR-125a-3p and miR-483-5p may jointly promote adipogenesis *in vivo* through regulating the RhoA/ROCK1/ERK1/2 pathway, at least in part.

## Discussion

**Figure 7:**
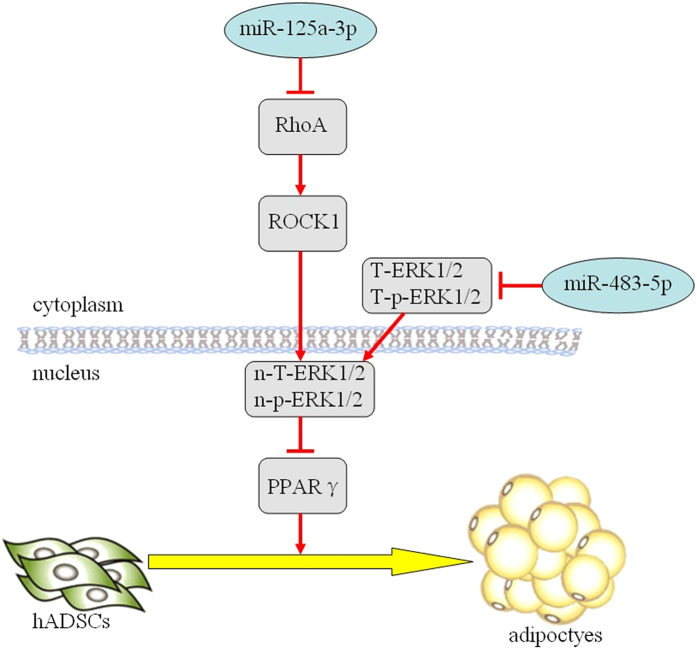
A proposed model of the regulation of adipogenesis by miR-125a-3p and miR-483-5p. In hADSCs, miR-125a-3p inhibits RhoA, resulting in decreased ROCK1 expression, which inhibits n-T-ERK1/2 and n-p-ERK1/2 in the nucleus. At the same time, miR-483-5p directly decreases T-ERK1/2 and T-p-ERK1/2 in the cytoplasm, and n-T-ERK1/2 and n-p-ERK1/2 in the nucleus. Subsequently, PPARγ, a downstream target of n-T-ERK1/2 and n-p-ERK1/2, which are negatively correlated with PPARγ, is increased and promotes adipogenesis.

In this study, we discovered that 18 miRs were upregulated in the SAT of MSL patients and that miR-125a-3p or miR-483-5p significantly promoted adipogenesis via the RhoA/ROCK1/ERK1/2 pathway (Fig. 7). Importantly, we found that miR-125a-3p and miR-483-5p promoted *de novo* adipose tissue formation in nude mice. These results demonstrated that miRs play an important role during adipogenesis and could be used in potential novel strategies for the prevention and treatment of MSL or obesity.

It has been reported that the m.8344A>G mutation in the tRNA^Lys^ is an important etiological factor of MSL^29^, but we did not find this mutation in the blood or SAT samples of our three MSL patients (data not shown). Therefore, we explored the functions of miRs in the pathogenesis of MSL. After miR array analysis, we found that 18 miRs were significantly upregulated. Although alcohol is believed to be an important pathogenic factor in MSL, none of the upreguated 18 miRs was correlated with alcohol addiction except of miR-140-3p^30^. Some of these 18 miRs have been reported to participate in adipogenesis. For example, let-7 represses adipogenesis in hMSCs and 3T3-L1 cells^31^,^32^; let-7e, let-7b, miR-28a-5p, and miR-10a are upregulated, while let-7c and miR-125b are downregulated in either preadipocytes or mature adipocytes of obese vs. lean SAT^33^. Moreover, miR-125b is directly and significantly correlated with body mass index^34^. In addition, miR-320 regulates insulin resistance in adipocytes^35^; miR-140 promotes adipogenesis^36^ and miR-483-3p inhibits 3T3-L1 adipogenesis and gradually decreases during differentiation^22^. However, the underlying molecular mechanisms of the regulation of adipogenesis by most of these miRs remain to be determined.

Our miR pathway analysis predicted that these upregulated miRs are closely related to the MAPK, Wnt, TGF-β, and regulation of actin cytoskeleton signaling pathways, which have been shown to negatively regulate adipogenesis^18^,^21^,^37^,^38^. These data suggest that miRs may participate in regulating these pathways and adipogenesis in MSL. Indeed, the miR-125a-3p and miR-483-5p agomir apparently promoted adipogenesis, while their antigomir significantly prevented adipogenesis under either induction or self-differentiation conditions. Previous studies have shown that miR-125a-3p and miR-483-5p target RhoA and ERK1, respectively^17^,^26^, which was further supported by our dual-luciferase assay.

miR-125a-5p, another mature sequence of miR-125a, has been found to be downregulated in the epididymal fat pads of leptin-deficient ob/ob mice. Interestingly, ssc-miR-125a has been found to repress the differentiation of porcine preadipocytes^15^. Consistent with the previous observation in mouse and human MSCs^16^, we found that miR-125a-3p expression was gradually upregulated in adipogenesis. A recent study has reported that miR-125a-3p is significantly related with fat mass and waist circumstance and that miR-125a-3p expression in the abdominal omental tissue in males is much higher than that in females^39^. Different from previous studies, our data showed that miR-125a-3p was highly expressed in the SAT of MSL patients, suggesting that miR-125a-3p may play different roles in MSL adipose tissue. miR-483-5p is located within the second intron of its host gene insulin-like growth factor 2 (IGF2) and was found to be coexpressed with IGF2 in 3T3-L1, Hepa1-6, and HepG2 cells^40^,^41^. It has been well documented that IGF2 promotes adipogenesis and is gradually upregulated during adipogenesis^42^,^43^. Our results demonstrated that miR-483-5p was also gradually upregulated during adipogenesis and promoted adipogenesis. These data suggest that miR-125a-3p and -5p as well as miR-483-3p and -5p may play reverse roles during adipogenesis, at least in adipogenesis of MSL patients.

RhoA and ERK1 are important negative regulation factors of adipogenesis, and dysregulation of their levels affects adipogenesis either in the stem cell or preadipocyte stage^20^,^21^,^24^. Our results demonstrated that the RhoA, ROCK1, T-ERK1/2, and T-p-ERK1/2 levels were downregulated in the SATs of MSL patients suggesting that downregulation of these negative regulation factors may promote adipogenesis in mature adipose tissue. We also found that RhoA, ROCK1, and T-p-ERK1/2, but not T-ERK1/2, were gradually downregulated during adipogenesis, consistent with previous studies^26^,^44^,^45^. It is unclear why upregulation of miR-483-5p decreased T-p-ERK1/2 but not T-ERK1/2 during adipogenesis. It remains to be determined whether other mechanisms participate in regulating T-ERK1/2 expression.

Several studies have demonstrated that the RhoA/ROCK pathway positively regulates p-ERK1/2 in the nucleus. For example, RhoA was found to activate n-p-ERK1/2 via ROCK^46^. Moreover, Brians *et al.* found that RhoA did not affect T-p-ERK but increased the nuclear localization of p-ERK^47^. Similarly, in vascular smooth muscle cells, alteration of ROCK1 and ROCK2 did not affect the T-ERK1/2, T-p-ERK1/2, or n-T-ERK1/2 expression levels but promoted nuclear translocation of n-p-ERK1/2^27^,^48^. However, a study using human leukemia cells showed that activation of the RhoA/ROCK1 pathway decreased p-ERK1/2 in the nucleus, and inhibition of RhoA/ROCK1 led to accumulation of n-p-ERK1/2^49^. Our data showed that miR-125a-3p did not affect T-ERK1/2 or T-p-ERK1/2, whereas it apparently altered the levels of n-T-ERK1/2 and n-p-ERK1/2, which was different from other previous studies. To determine whether miR-483-5p regulates the RhoA/ROCK1/ERK1 pathway, we compared the expression levels of these proteins in hADSCs transfected with the miR-125a-3p inhibitor, miR-483-5p mimic, or cotransfection of the miR-125a-3p inhibitor and miR-483-5p mimic, and found that cotransfection did not change the T-ERK1/2 or T-p-ERK1/2 levels but apparently changed the n-T-ERK1/2 and n-p-ERK1/2 levels. Furthermore, cotransfected with the miR-125a-3p agomir and pCEP4L-ERK1 partially restored adipogenesis compared to pCEP4L-ERK1, and similar protein expression of PPARγ and C/EBPα were observed. These data further supported the conclusions that miR-125a-3p affects the T-ERK1/2 and p-ERK1/2 levels in the nucleus and that miR-125a-3p and miR-483-5p may jointly regulate the RhoA/ROCK1/ERK1/2 pathway.

Accumulating data demonstrate that miRs promote *de novo* adipogenesis *in vivo*. For example, LV-miR-637 and miR-146b regulate adipogenesis^50^,^51^. We found that LV-miR-125a, LV-miR-483, and especially LV-miR-125a in combination with LV-miR-483 significantly increased cells size and promoted *de novo* fat formation *in vivo* and *de novo* adipose tissue weight gain. Similar to the *in vitro* experiments, miR-125a did not affect T-ERK1/2 or T-p-ERK1/2 expression but decreased RhoA and ROCK1 expression, accompanied with decreased n-T-ERK1/2 and n-p-ERK1/2 expression. LV-miR-125 + 483 further decreased the n-T-ERK1/2 and n-p-ERK1/2 levels. Thus, miR-125a-3p and miR-483-5p regulate the activity of the RhoA/ROCK1/ERK1/2 pathway *in vivo*.

In summary, we found that both miR-125a-3p and miR-483-5p are significantly increased in the SATs of MSL patients and that miR-125a-3p and miR-483-5p promote adipogenesis through regulating the RhoA/ROCK1/ERK1/2 pathway. Although we were not allowed to get enough tissues to isolate hADSC from MSL patients, our findings suggest that the RhoA/ROCK1/ERK1/2 signaling pathway is a potential therapeutic target for the development of drugs that prevent or treat MSL or obesity patients.

## Additional Information

**How to cite this article**: Chen, K. *et al.* miR-125a-3p and miR-483-5p promote adipogenesis via suppressing the RhoA/ROCK1/ERK1/2 pathway in multiple symmetric lipomatosis. *Sci. Rep.* **5**, 11909; doi: 10.1038/srep11909 (2015).

## Supplementary Material

Supplementary Information

## Figures and Tables

**Table 1:** Anthropometric and metabolic characteristics of the study participants.

**Characteristics**	**Controls (n = 3)**	**MSL (n = 3)**
Age (y)	54.2 ± 3.7	50.5 ± 4.5
BMI (kg/m^2^)	26.9 ± 0.81	27.13 ± 0.64
Cholesterol (mmol/l)	3.92 ± 0.82	4.91 ± 1.08
TG (mmol/l)	1.62 ± 0.56	1.93 ± 0.41
LDL (mmol/l)	2.8 ± 0.29	1.75 ± 0.37
HbA1c (%)	6.03 ± 0.42	5.21 ± 0.37
FBS (mmol/l)	5.4 ± 0.3	5.2 ± 0.5
Total body adipose tissue/total body tissue	36.29 ± 3.94	46.94 ± 5.32*
Abdominal SCAT area (cm2)	120.8 ± 27.8	260.2 ± 20.3*

^*^p < 0.05 vs. controls. BMI: body mass index; TG: Triglycerides; LDL: low-density lipoprotein; FBS: fasting plasma glucose.

**Table 2:** Upregulated miRs in the SAT of MSL patients compared to control group.

**miR Name**	**P-Value**	**Fold Change**
hsa-let-7b-3p	0.032934649	27.2052843
hsa-let-7e-3p	0.03501016	26.84184621
hsa-miR-629-3p	0.03201299	20.72297174
hsa-miR-483-5p	0.011343835	17.48972864
hsa-miR-574-3p	0.01307515	16.63161544
hsa-miR-10a-3p	0.026807951	12.81106936
hsa-let-7c-5p	0.035804091	12.37382817
hsa-miR-28-3p	0.035720409	11.31250089
hsa-miR-196a-3p	0.016357114	9.552199712
hsa-miR-125b-5p	0.011139626	9.507005532
hsa-miR-197-5p	0.011205909	9.373410074
hsa-miR-125a-3p	0.010228823	8.971728732
hsa-miR-320b	0.013699372	8.858724555
hsa-miR-625-3p	0.01936573	8.35511682
hsa-miR-339-3p	0.038850611	5.881148829
hsa-miR-140-3p	0.008839799	4.290066467
hsa-miR-432-5p	0.012643956	3.73105855
hsa-miR-625-3p	0.029979201	3.628931941

The relative miR expression in the SAT of MSL patients compared to controls were detected by TaqMan miR array analys.
