# Supplementary material for: miR-125a-3p and miR-483-5p promote adipogenesis via suppressing the RhoA/ROCK1/ERK1/2 pathway in multiple symmetric lipomatosis
Source: Sci Rep. 2015 Jul 7;5:11909. doi: 10.1038/srep11909 (PMC4493643; doi:10.1038/srep11909)

**miR-125a-3p and miR-483-5p promote adipogenesis via suppressing the RhoA/ROCK1/ERK1/2 pathway in multiple symmetric lipomatosis**

**Ke Chen, Honghui He, Yanhong Xie, Liling Zhao, Shaoli Zhao, Xinxing Wan, Wenjun Yang & Zhaohui Mo***

Department of Endocrinology, Third Xiangya Hospital of Central South University, Changsha, Hunan, 410013, China

Correspondence and requests for materials should be addressed to Z.H.M.(easd04mzh@126.com)

full-length blots gels are presented in Supplementary Figure 2-D


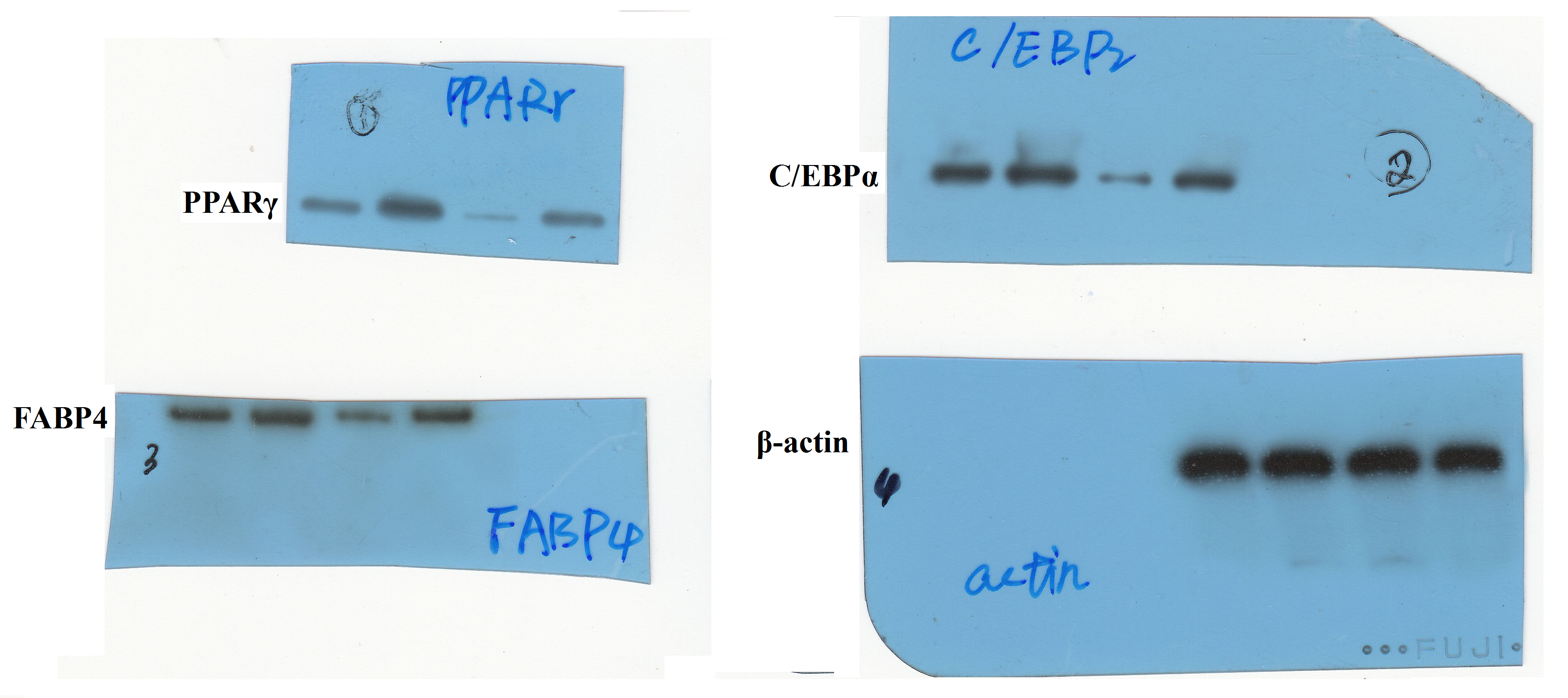


full-length blotsgels are presented in Supplementary Figure 2-E


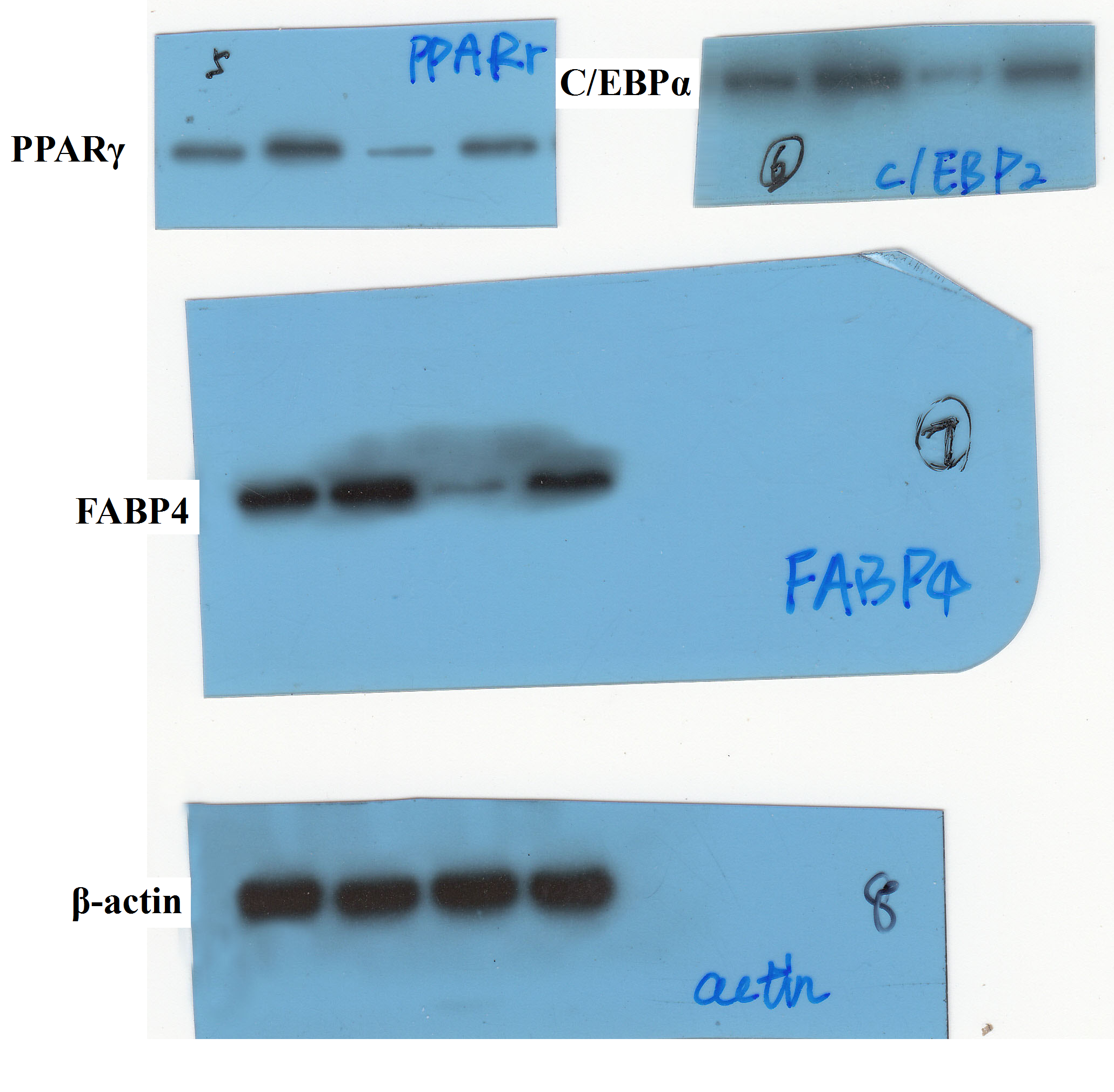


full-length blotsgels are presented in Supplementary Figure 3-C


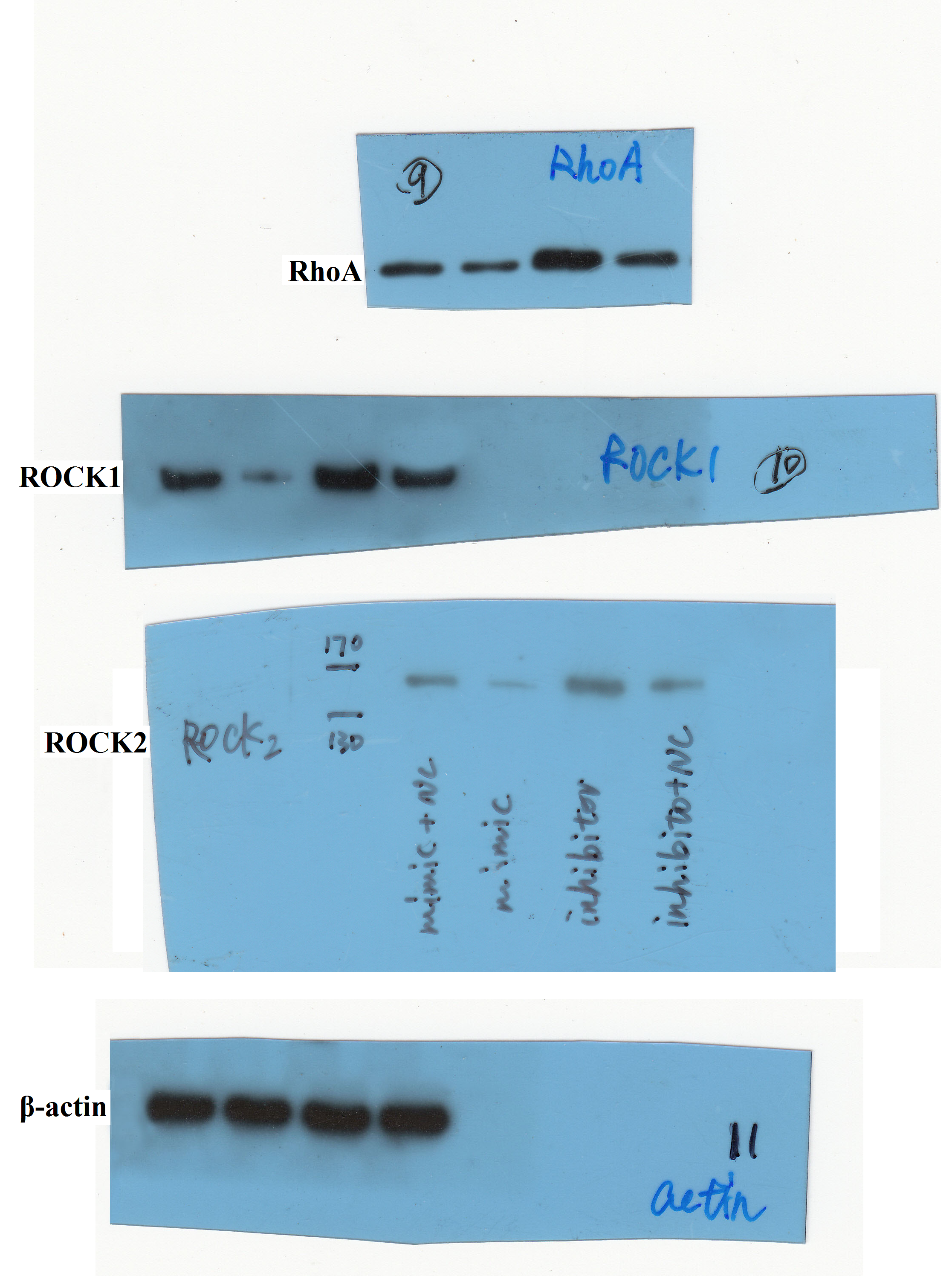


full-length blotsgels are presented in Supplementary Figure 3-D


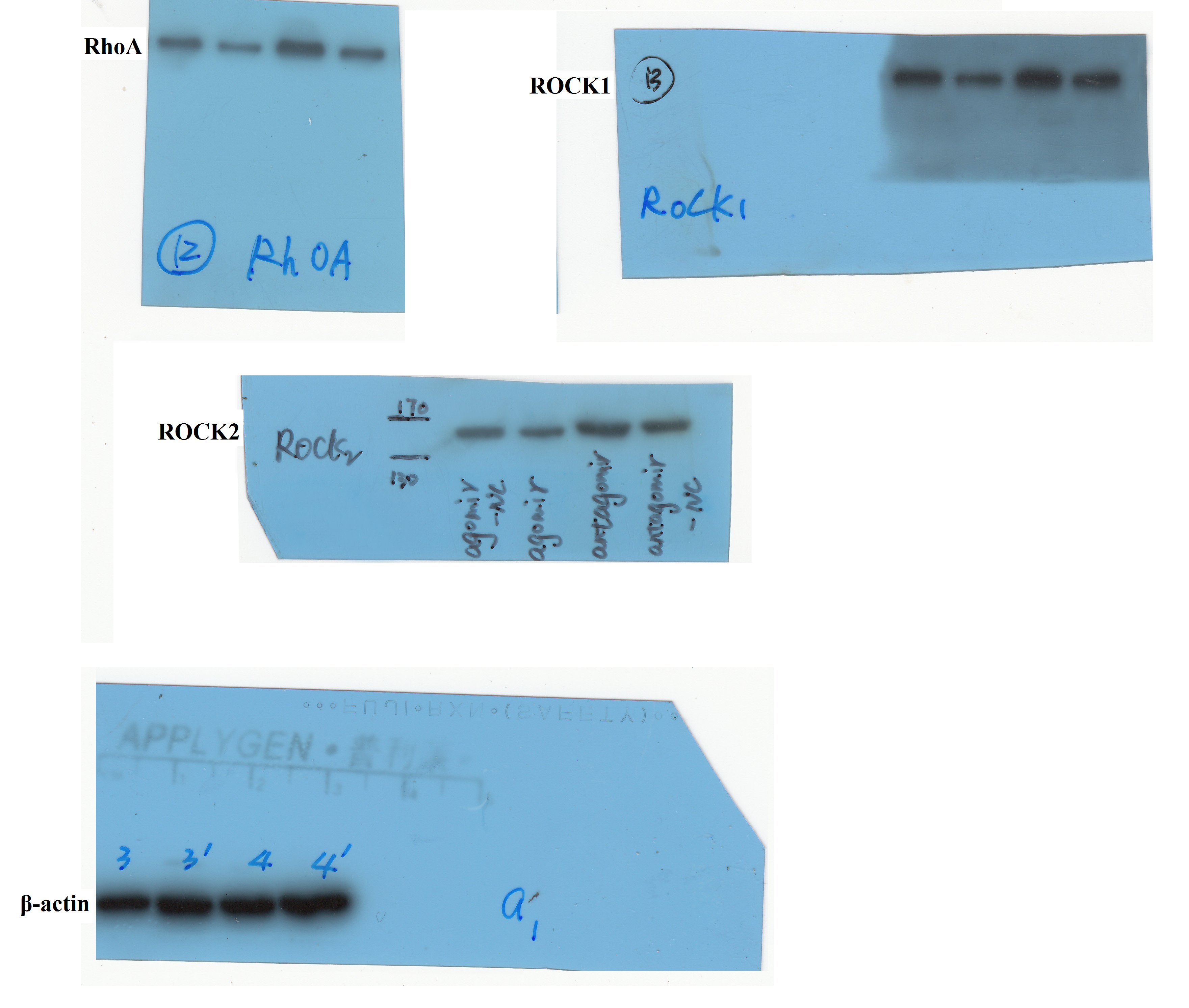


full-length blotsgels are presented in Supplementary Figure 3 E and F


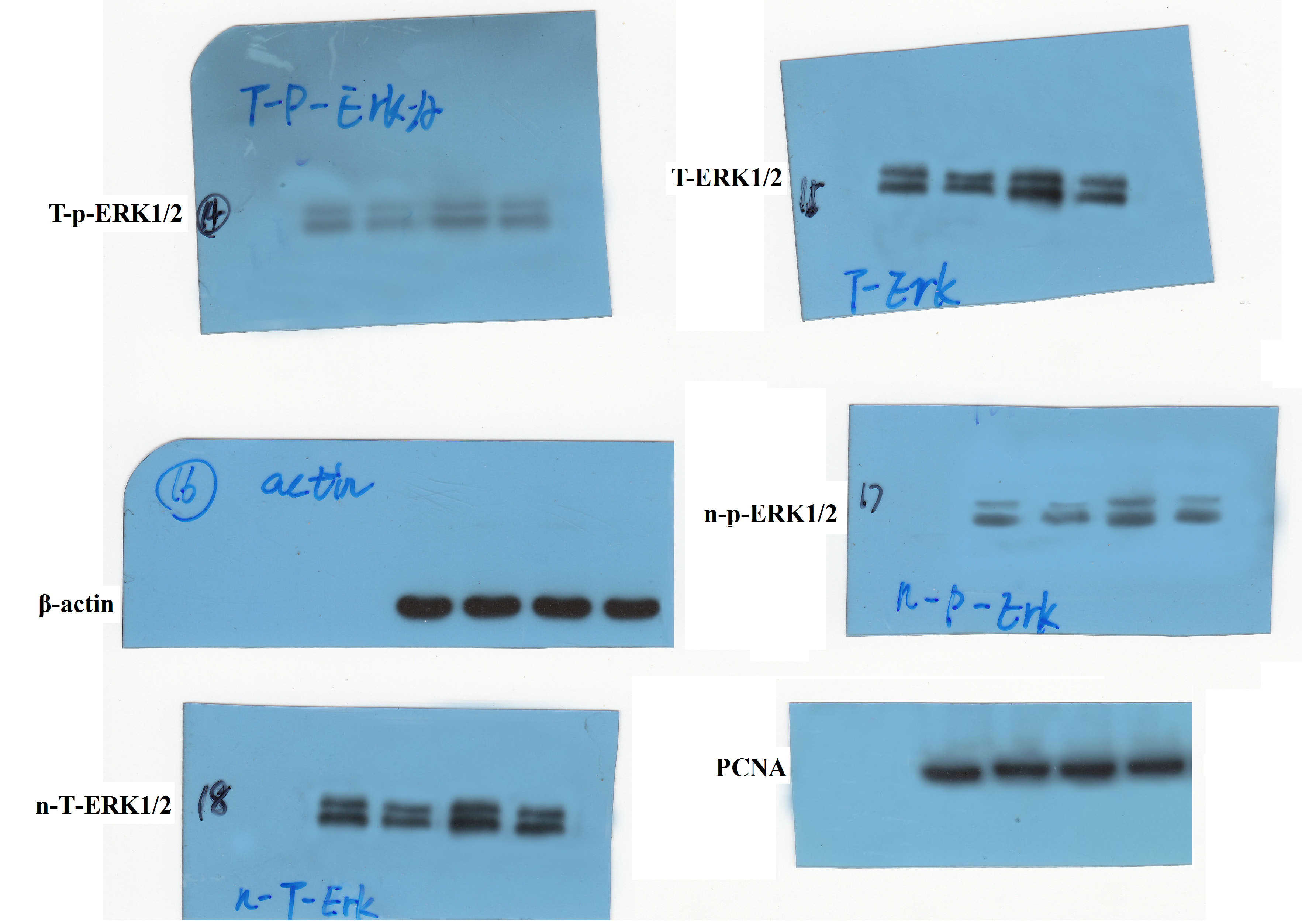


full-length blotsgels are presented in Supplementary Figure 4-B


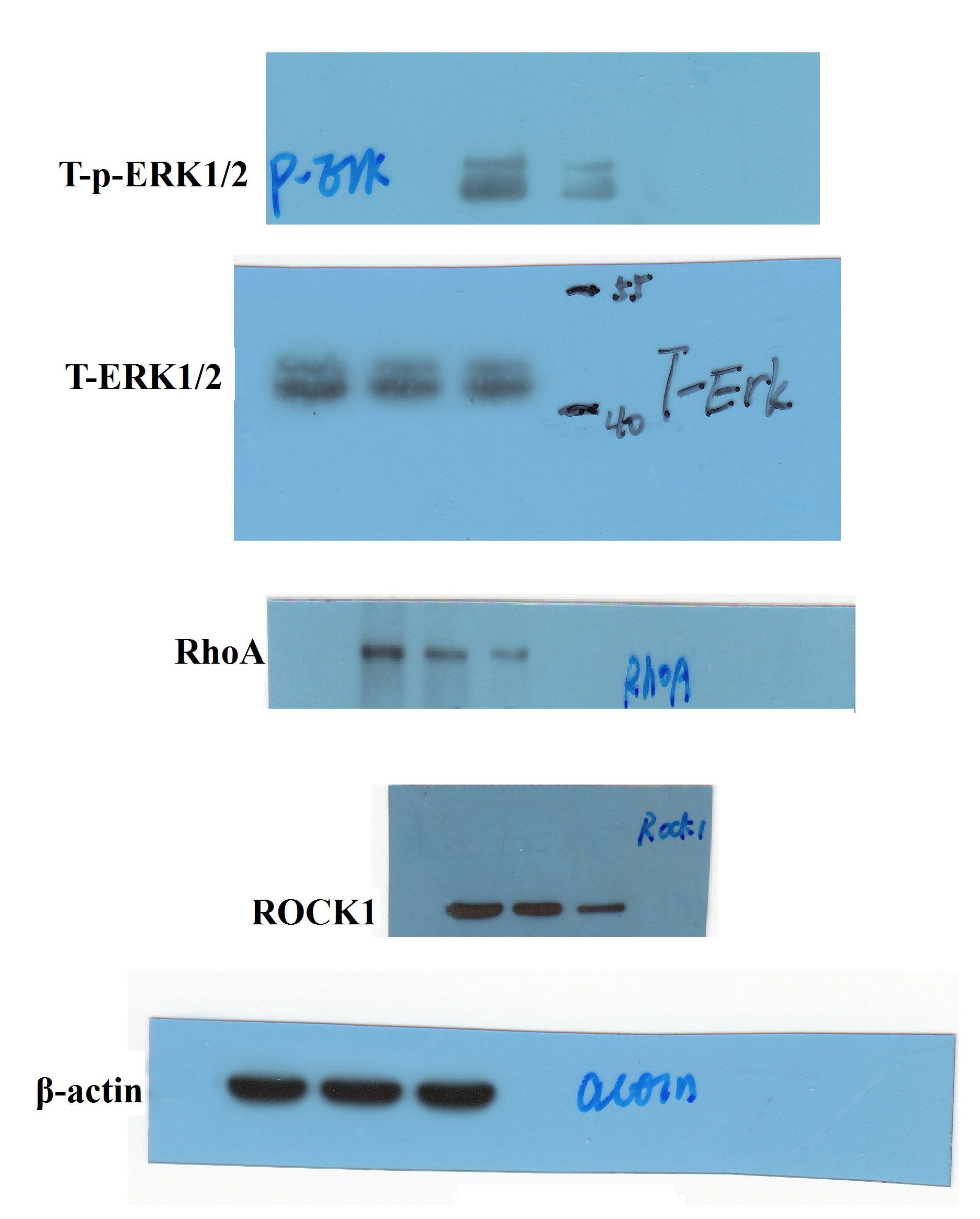


full-length blotsgels are presented in Supplementary Figure 4-C


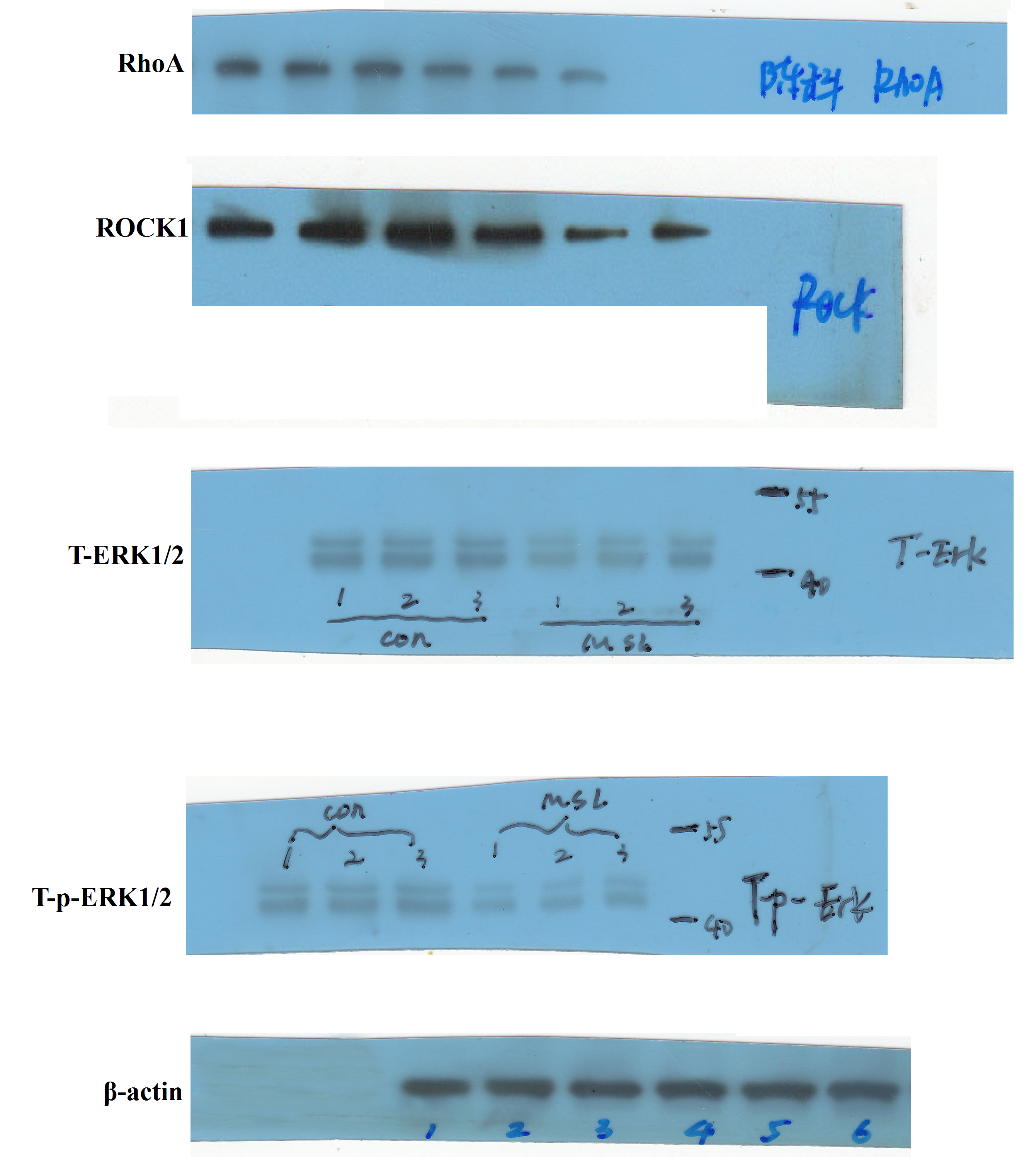


full-length blotsgels are presented in Supplementary Figure 5-A


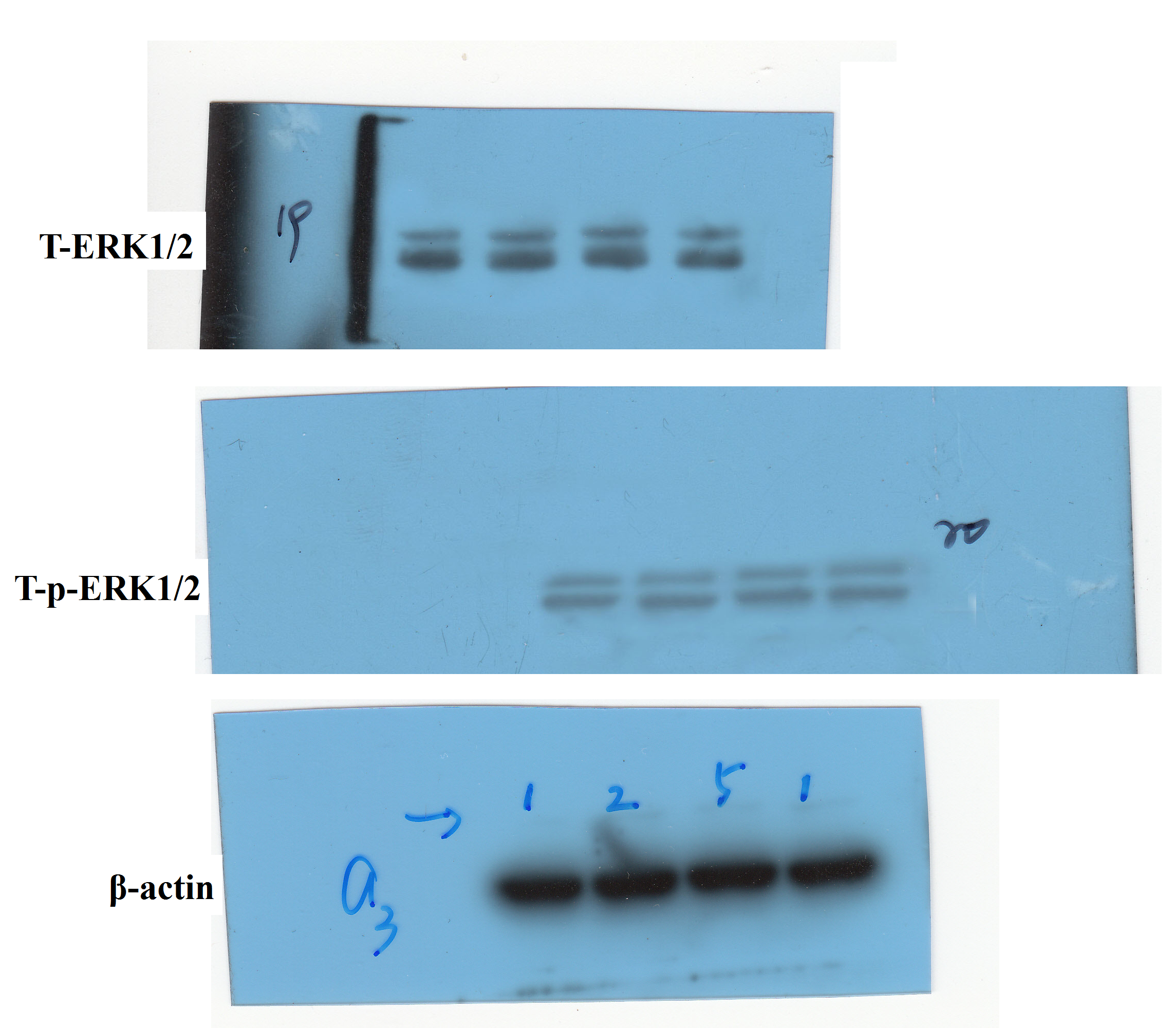


full-length blotsgels are presented in Supplementary Figure 5-B


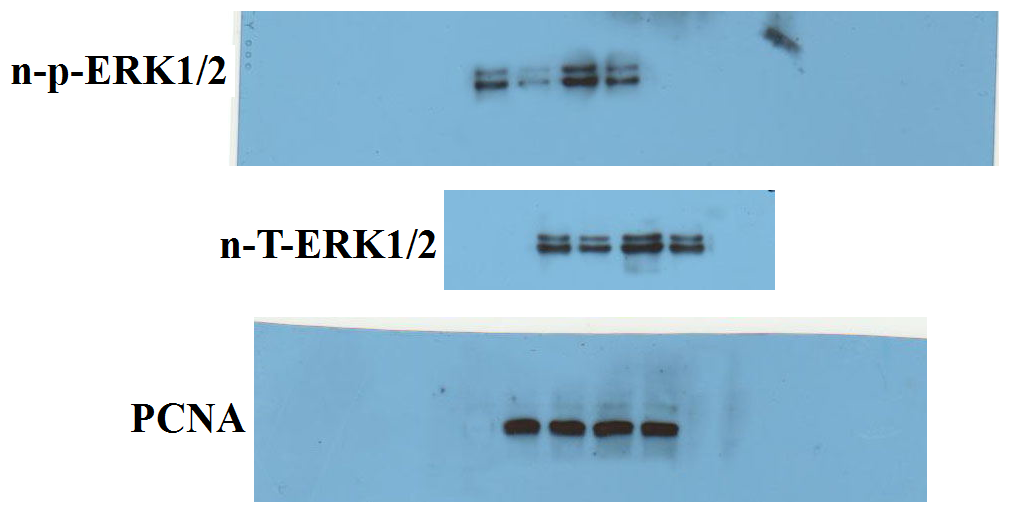


full-length blotsgels are presented in Supplementary Figure 5-C


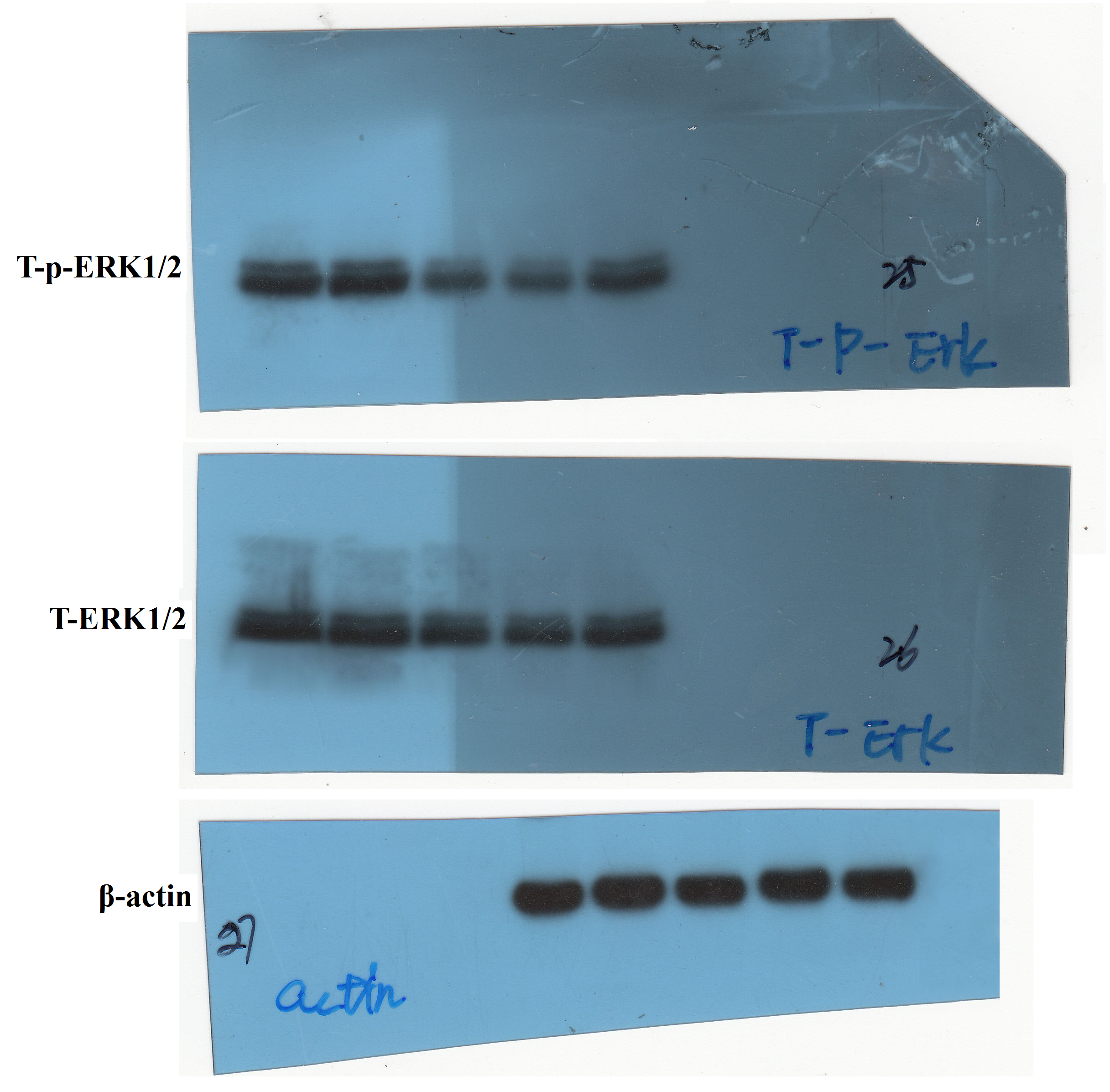


full-length blotsgels are presented in Supplementary Figure 5-D


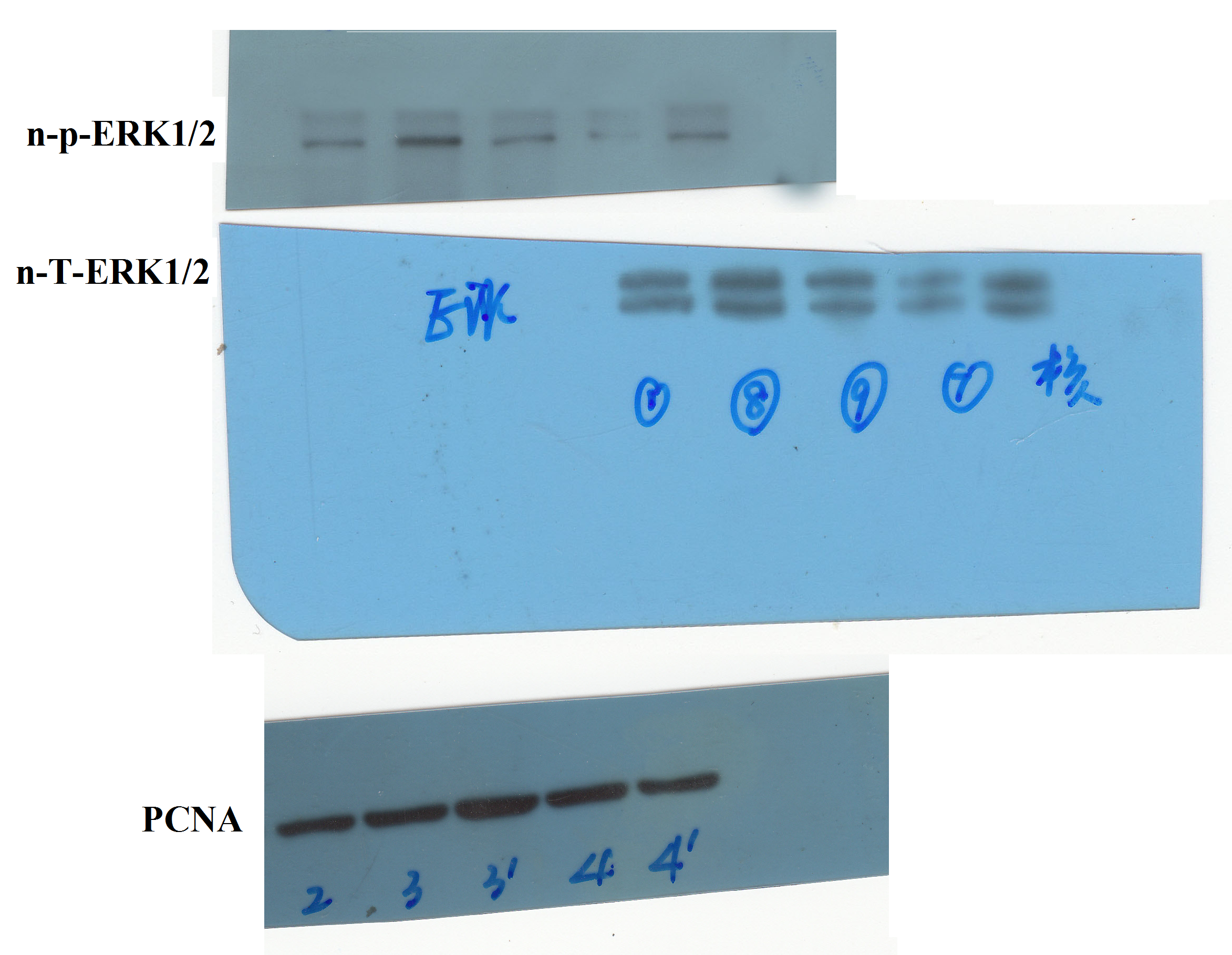


full-length blotsgels are presented in Supplementary Figure 5-F


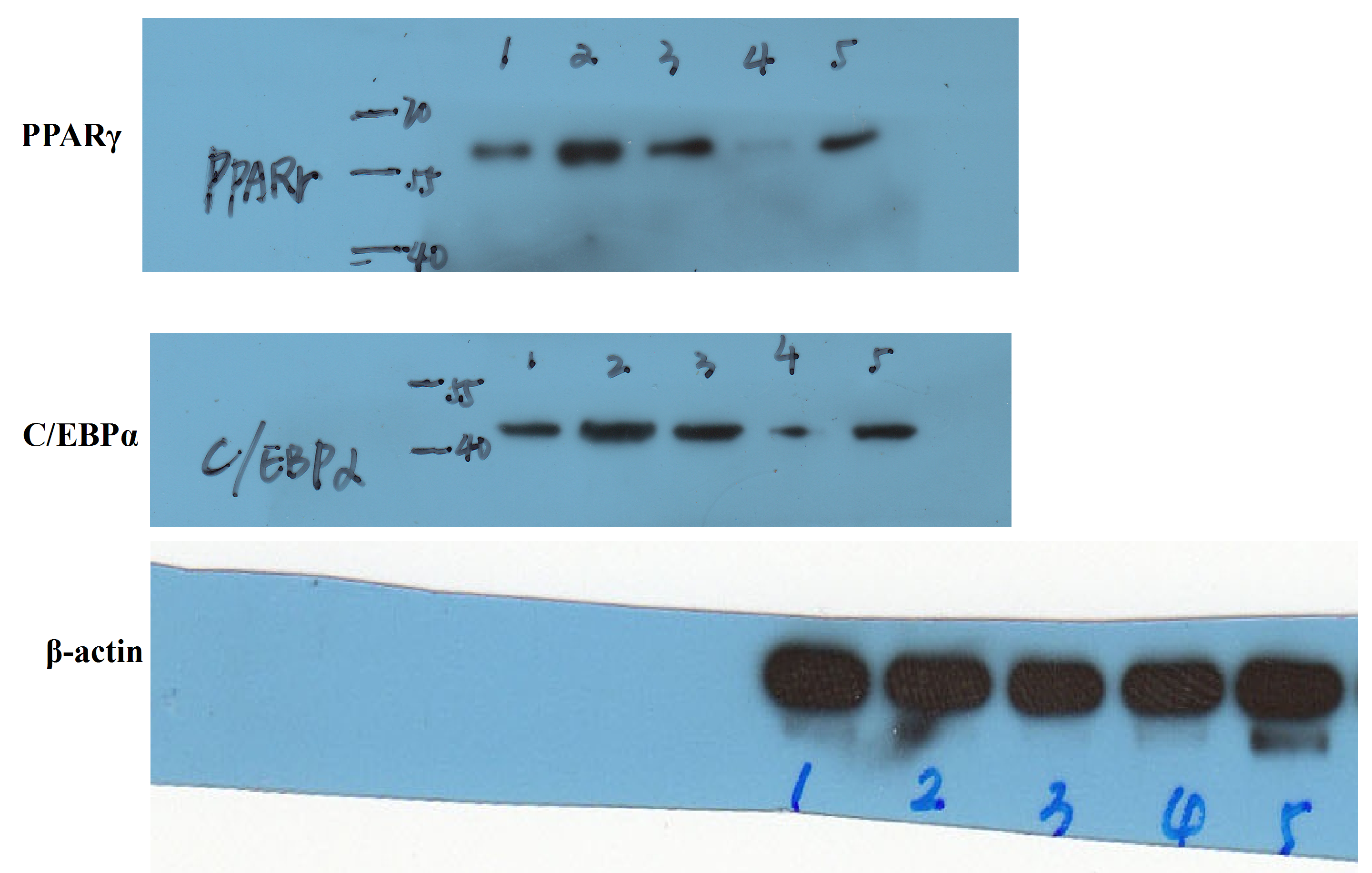


full-length blotsgels are presented in Supplementary Figure 6-F


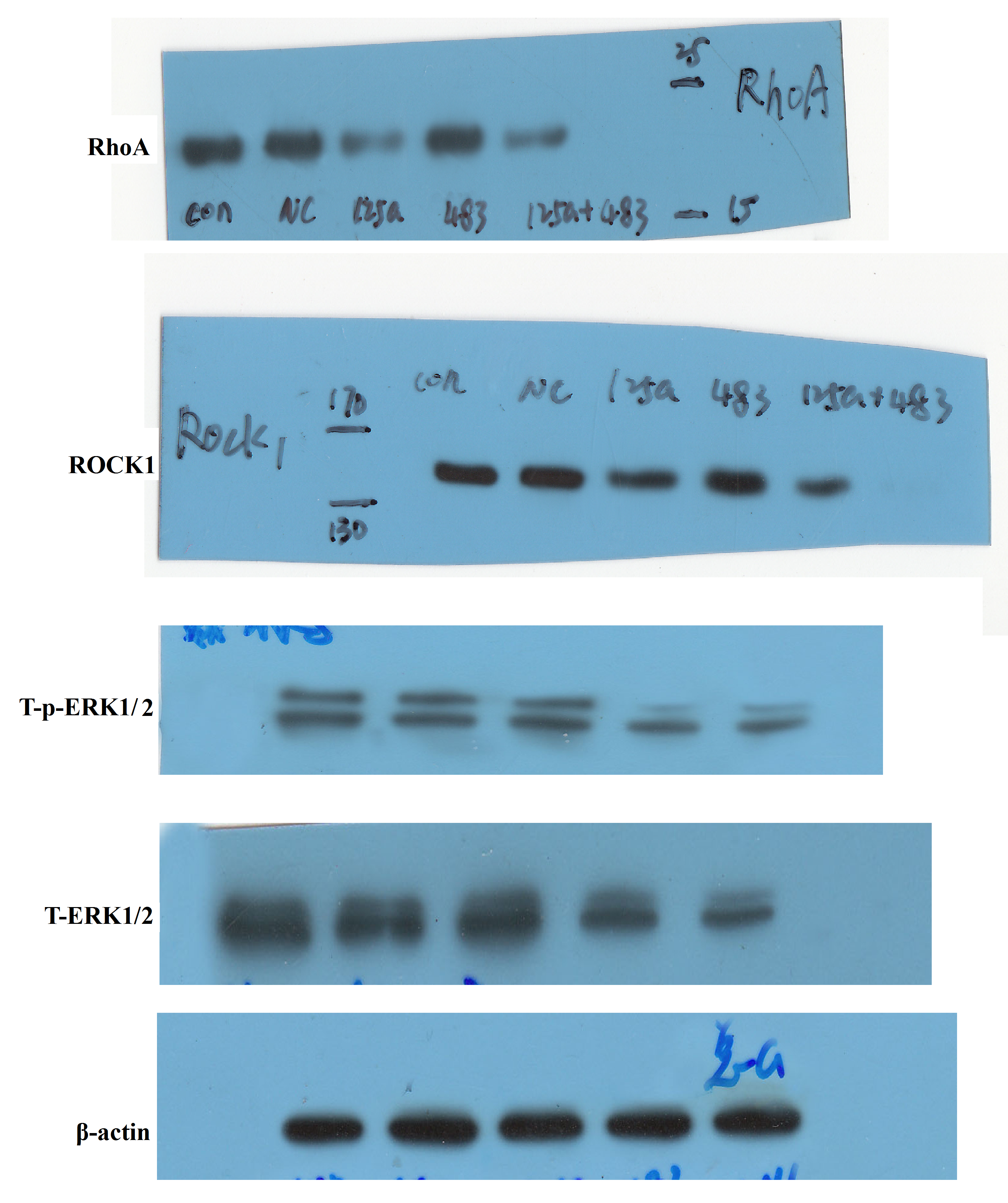


full-length blotsgels are presented in Supplementary Figure 6-G


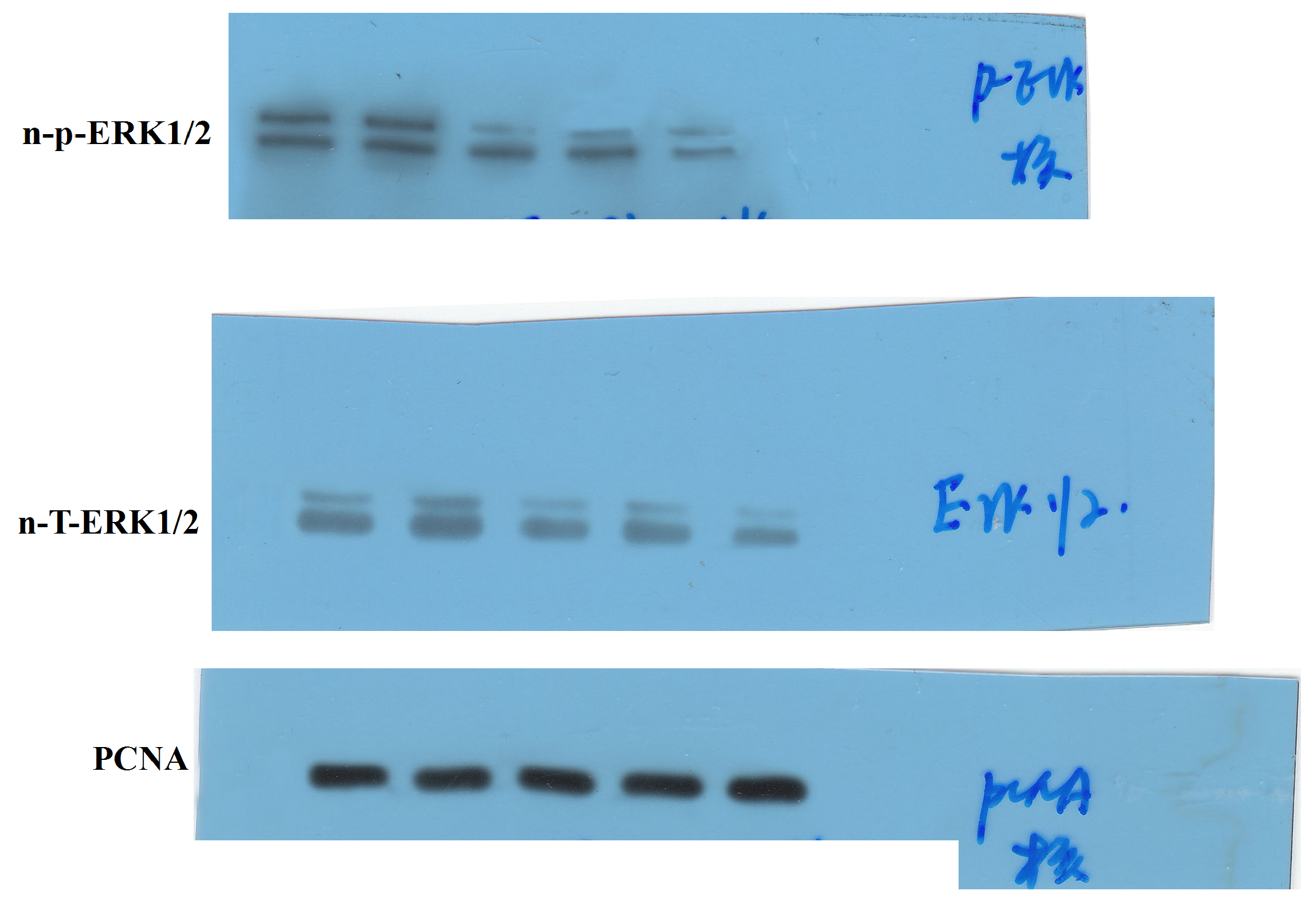

Supplement: Supplementary Information [file srep11909-s1.doc]
